# Influences of pH and Iron Concentration on the Salivary Microbiome in Individual Humans with and without Caries

**DOI:** 10.1128/AEM.02412-16

**Published:** 2017-02-01

**Authors:** Jianye Zhou, Nan Jiang, Zhenzhen Wang, Longqing Li, Jumei Zhang, Rui Ma, Hongbing Nie, Zhiqiang Li

**Affiliations:** a Key Laboratory of Oral Diseases of Gansu Province, Key Laboratory of Stomatology of the State Ethnic Affairs Commission, Northwest University for Nationalities, Lanzhou, Gansu, China; b Institute of Applied Ecology, Chinese Academy of Sciences, Shenyang, China; c School of Stomatology, Lanzhou University, Lanzhou, China; d Institute of Modern Physics, Chinese Academy of Sciences, Lanzhou, China; University of Calgary

**Keywords:** caries, iron, microbial communities, pH, saliva

## Abstract

This study aimed to identify the differences in the oral microbial communities in saliva from patients with and without caries by performing sequencing with the Illumina MiSeq platform, as well as to further assess their relationships with environmental factors (salivary pH and iron concentration). Forty-three volunteers were selected, including 21 subjects with and 22 without caries, from one village in Gansu, China. Based on 966,255 trimmed sequences and clustering at the 97% similarity level, 1,303 species-level operational taxonomic units were generated. The sequencing data for the two groups revealed that (i) particular distribution patterns (synergistic effects or competition) existed in the subjects with and without caries at both the genus and species levels and (ii) both the salivary pH and iron concentration had significant influences on the microbial community structure.

**IMPORTANCE** The significant influences of the oral environment observed in this study increase the current understanding of the salivary microbiome in caries. These results will be useful for expanding research directions and for improving disease diagnosis, prognosis, and therapy.

## INTRODUCTION

Dental caries represent a chronic infectious disease with the highest incidence among human oral diseases and a wide distribution (1). In most developed and developing countries, caries are a serious health problem that affect a large proportion of children and adults (2). Therefore, the early diagnosis and prevention of dental caries is important in the 21st century (3).

The oral microbial community structure, rather than a single bacterial species, has been reported to have a stronger influence on oral health, including caries. The structure of the oral microbial community, which includes a range of structural and functional configurations (4), changes in the abundance of certain taxa (5, 6), and the cooccurrence of certain microbes (7), is closely related to caries. Moreover, relationships exist between the microbial communities and many factors in the oral environment (8, 9). Several potential factors have been reported to have significant relationships with caries-associated bacteria, as well as with microbial homeostasis (9–12). For example, intraoral pH has been reported to have a strong effect on the structure of microbial communities, especially for partial dentin caries-associated microbiota, such as some Lactobacillus species (12–14). Alternatively, salivary iron, an important elemental metal in saliva, could provide essential nourishment for oral bacterial species (15), and it has been shown to modulate the salivary microbial profile (16). However, the intraoral pH varies (14), and related studies of iron have been mainly based on *in vitro* experiments (16). Moreover, there is no report of the influence of iron on the microbial community structure in human saliva in patients with and without caries. Thus, more investigations are necessary to determine the clinical significance of the two factors.

In this study, 43 salivary microbial communities were sequenced using the Illumina MiSeq platform. To minimize the effects of the subjects' living environments on the oral microbial communities (9, 17), volunteers with similar living environments and habits were selected from Meipo Village in Jishi Shan, Gansu, China. The influences of oral environmental factors, such as pH and iron concentration, on the bacterial community structure were investigated to determine the role of the microbiome in caries. In the present study, an influence of iron on the microbial community structure in human saliva from patients with and without caries was detected, and salivary pH was synchronously analyzed. The results of this study could increase the current understanding of the correlations between the oral microbiome and caries; this information will be useful for the rapid diagnosis of diseases, for predicting patient prognosis, and for monitoring the targetability and efficacy of therapy for caries.

## RESULTS

### Similar bacterial diversities in salivary samples from patients with and without caries.

After parallel sequencing of the 43 salivary samples, a total of 966,255 trimmed sequences were obtained, with 13,749 normalized reads per microbiome. According to clustering at the 97% similarity level, 1,303 species-level operational taxonomic units (OTUs) were generated, of which 95.2% were shared by the subjects with and without caries. α-Diversity based on four indices did not significantly differ between the subjects with and without caries (*P* > 0.05). A β-diversity comparison indicated that the salivary samples from the patients with and without caries exhibited similar bacterial community structures (see Fig. S1 in the supplemental material).

**FIG 1:**
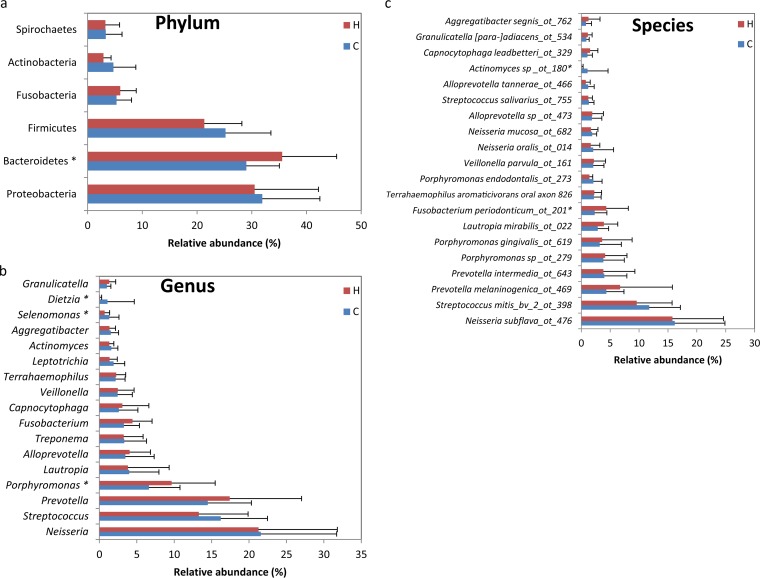
Comparison of bacterial taxonomy (>1% relative abundance) of samples from individuals with (C) and without (H) caries at the phylum (a), genus (b), and species (c) levels. *, *P* < 0.05.

**FIG 2:**
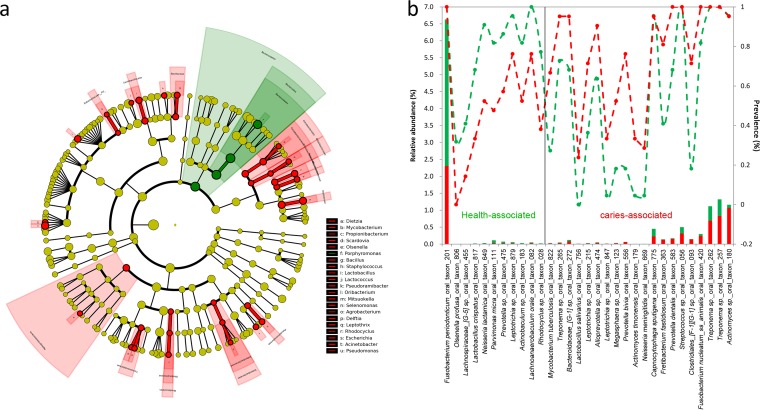
Non-caries- and caries-associated genera (a) and species (b), as determined based on the LEfSe method. The nonparametric factorial Kruskal-Wallis rank sum test was used to detect the taxa with significant differential relative abundance between the healthy group and the caries group at a significance level of 0.05. The relative abundance (the histograms) and prevalence (the dotted lines) of each species are also shown in panel b. Green and red indicate data from healthy and caries groups, respectively.

The OTUs were assigned to 12 phyla, and more than 99% belonged to six phyla (>1% relative abundance), including Proteobacteria, Bacteroidetes, Firmicutes, Fusobacteria, Actinobacteria, and Spirochaetes (Fig. 1a). A total of 21 classes, 35 orders, 72 families, 145 genera, and 415 species were identified, of which only 9, 11, 15, 17, and 20 members, respectively, were predominant (>1% relative abundance) at each taxonomic level (Fig. 1 and Fig. S2). Within the main taxa (>1% relative abundance), the non-caries-associated taxa based on differential distribution (*P* < 0.05) included the phylum Bacteroidetes (Fig. 1a), the class Bacteroides (Fig. S2a), the order Bacteroidales (Fig. S2b), the genus Porphyromonas (Fig. 1b), and the species Fusobacterium periodonticum oral taxon 201 (4.31% and 2.31% for the H and C groups, respectively) (Fig. 1c), while the family Dietziaceae (Fig. S2c), the genera Dietzia and Selenomonas (Fig. 1b), and the species Actinomyces sp. strain oral taxon 180 (0.09% and 1.07% for the H and C groups, respectively) (Fig. 1c) were associated with caries. Another 53 caries-associated minor taxa (<1% relative abundance), including one class, four orders, 11 families, 18 genera, and 19 species, and 10 non-caries-associated species were also found (Fig. 2a and b). The relative abundance and prevalence of each non-caries- and caries-associated species are further shown in Fig. 2b. Among these species, for example, Olsenella profusa oral taxon 806 was detected only in healthy individuals at a low relative abundance (0.003%) but with a prevalence of over 31%. In addition, we compared our results with those of previous studies comparing individuals with and without caries, and the overlapping genera and species are listed in Table S2; discrepancies among the results were due to differences in the sample types, sampling positions, and methods used.

### Different cooccurrence patterns in subjects with and without caries.

**FIG 3:**
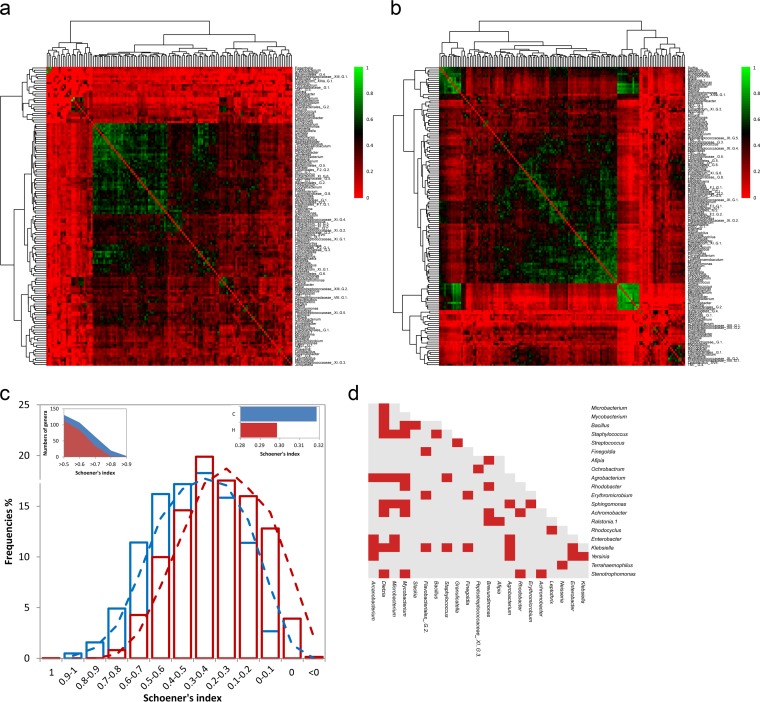
Heatmaps displaying genus distribution patterns for the healthy (a) and caries (b) groups. Increasing values are translated into colors from red to green. Trees were clustered based on the similarity of the Schoener's values. (c) The cooccurrence probabilities based on the Schoener's index were divided into 13 intervals, including <0, 0, 0 to 0.1, 0.1 to 0.2, 0.2 to 0.3, 0.3 to 0.4, 0.4 to 0.5, 0.5 to 0.6, 0.6 to 0.7, 0.7 to 0.8, 0.8 to 0.9, 0.9 to 1, and 1. The frequency of the Schoener's index falling into each interval was calculated in both the caries group and the healthy group. The number of involved genera at the different intervals and a global comparison of the Schoener's index between the two groups (using a *t* test) are also shown within the plot. (d) All pairs with Schoener's index greater than 0.8.

Schoener's cooccurrence index (abundance-based) was calculated for each pair of genera, and heatmaps were generated to separately assess the distribution patterns (Fig. 3). In each group, all genera were gathered into two clusters from the root of the dendrogram based on the similarities of their cooccurrence probabilities, but the assignments in the subcluster were more unbalanced in the caries group than those in the healthy group (Fig. 3a and b). In addition, higher cooccurrence probabilities (Schoener's index of >0.5) were more frequently observed in the subjects with caries than in the healthy subjects (Fig. 3c). For example, substantial cooccurrence probabilities (>0.8) were only detected between Microbacterium and Agrobacterium and between Eggerthella and Achromobacter, whereas a total of 54 pairs, covering 29 genera, had cooccurrence probabilities of greater than 0.8 in the individuals with caries (Fig. 3d). These data indicated that varied distribution patterns existed in the subjects with and without caries.

However, at the species level, similar distribution patterns were detected in the two groups (Fig. S3a), although the genera harboring higher cooccurrence probabilities (Schoener's index of >0.8) were still more prevalent in the caries group than in the healthy group (Fig. S3b).

### Salivary pH has a negative relationship with iron concentration.

**FIG 4:**
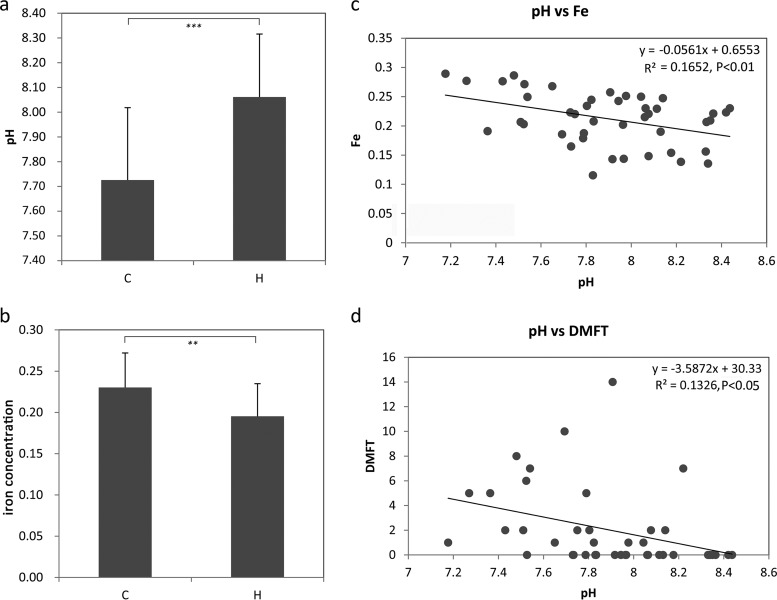
Salivary pH and iron concentration. Significant differences in the salivary pH (a) and iron concentration (b) between samples from individuals with (C) and without (H) caries. ***, *P* < 0.001; **, *P* < 0.01. (c) Significant correlations between the salivary pH and iron concentration. (d) Significant correlations between the salivary pH and DMFT index.

Both the pH and iron concentration significantly differed between the subjects with and without caries (*P* < 0.001) (Fig. 4a and b). A significant negative relationship between the salivary pH and iron concentration was observed in the 43 individuals (Fig. 4c). However, only the salivary pH was significantly correlated with the decayed, missing, and filled teeth (DMFT) index (Fig. 4d).

### Influence of important variables on bacterial community structure.

**FIG 5:**
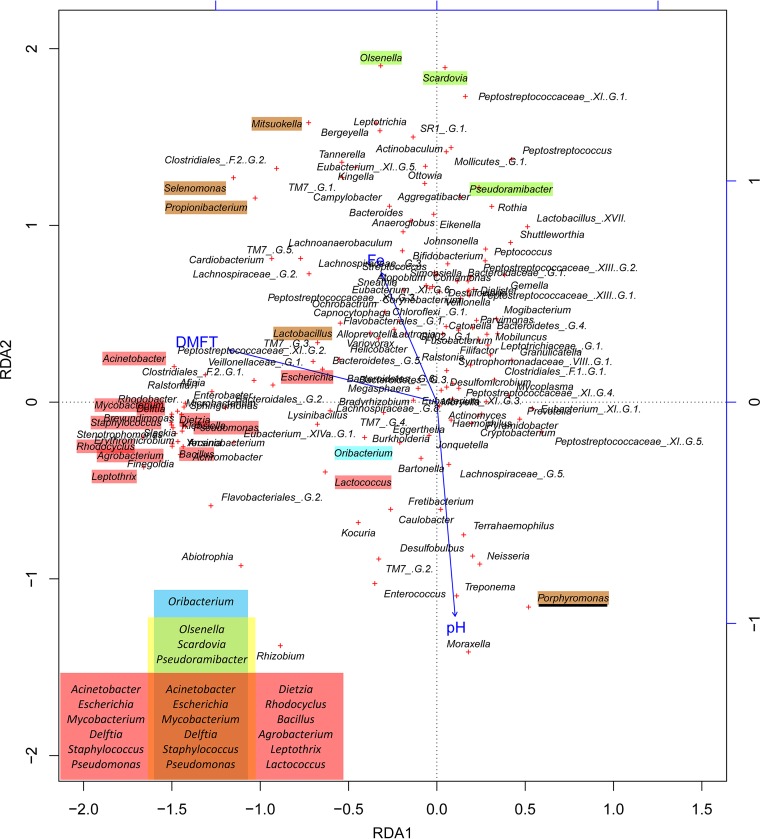
Redundancy analysis (RDA) of the significant influences of the salivary pH, iron concentration, and DMFT index on the bacterial community structure at the genus level. Colors indicate the non-caries-associated (underlined) and caries-associated genera. These distinct genera with high correlations with one or more variables are explained by the Venn diagram inserted in the plot: yellow, blue, and red indicate genera with high correlations (>0.6) with iron concentration, pH, and DMFT, respectively.

The salivary pH, oral iron concentration, and DMFT index significantly influenced the bacterial community structure at the genus and species levels (*P* < 0.01) (Fig. 5). The redundancy analysis (RDA) plots showed that the caries- and non-caries-associated genera could be divided into two groups: those highly related to one variable (e.g., Oribacterium with salivary pH), or to more than one variable, and those clustered near the DMFT arrow (Fig. 5). As shown in the Venn diagram in Fig. 5, in addition to the genus Oribacterium, briefly, a cluster of 12 caries-associated genera was correlated with the DMFT. Three other caries-associated genera, including Olsenella, Scardovia, and Pseudoramibacter, showed high correlations with pH and iron, and the others, including four caries- and one non-caries-associated genera, were highly related to all three variables. However, the correlations between the factors and species were more complex (Fig. S4), and the species exhibiting strong correlations are further listed in Table 1.

**TABLE 1:** Details for species with high correlations with the three variables^*f*^

Variable and no.^*a*^	Phylum	Species	HOT^*b*^
Positive			
pH			
S40	Bacteroidetes	Capnocytophaga sp.	332
S269^*e*^	Bacteroidetes	Prevotella multisaccharivorax	794
**S227**	Bacteroidetes	Prevotella oralis	705
**S45**	Bacteroidetes	Alloprevotella sp.	912
**S382**^*e*^	Firmicutes	Streptococcus sobrinus	768
S158	Fusobacteria	Leptotrichia buccalis	563
S1	Proteobacteria	Neisseria subflava	476
**S154**	Proteobacteria	Cardiobacterium hominis	633
**S237**^*c*^	Proteobacteria	Neisseria lactamica	649
S344	Spirochaetes	Treponema sp.	227
**S306**	Tenericutes	Mycoplasma faucium	606
Iron			
**S43**	Bacteroidetes	Prevotella sp.	299
**S116**	Bacteroidetes	Prevotella veroralis	572
S194	Bacteroidetes	Capnocytophaga sp.	863
S330^*e*^	Firmicutes	Lachnoanaerobaculum sp.	496
**S207**	Firmicutes	Mitsuokella sp.	521
**S218**	Firmicutes	Moryella sp.	419
**S46**	Firmicutes	Streptococcus constellatus	576
S60^*d*^	Firmicutes	Streptococcus sp.	56
**S351**	Proteobacteria	Desulfovibrio sp.	40
S38	Spirochaetes	Treponema amylovorum	541
**S123**^*d*^	Synergistetes	Fretibacterium fastidiosum	363
DMFT			
**S183**	Actinobacteria	Atopobium rimae	750
S310^*e*^	Bacteroidetes	Capnocytophaga sp.	902
S125	Bacteroidetes	Prevotella shahii	795
**S234**^*d*^	Firmicutes	Megasphaera sp.	123
S181	Firmicutes	Lachnospiraceae [G-2] sp.	88
S120^*d*^	Firmicutes	Clostridiales [F-1][G-1] sp.	93
**S109**	Firmicutes	Veillonellaceae [G-1] sp.	155
S54	Spirochaetes	Treponema denticola	584
**S352**^*e*^	TM7	TM7 [G-4] sp.	355
Negative			
pH			
S141	Bacteroidetes	Bacteroidetes [G-6] sp.	516
S98	Bacteroidetes	Bacteroidetes [G-5] sp.	505
S14	Bacteroidetes	Porphyromonas endodontalis	273
**S256**	Bacteroidetes	Bacteroidetes [G-5] sp.	511
S51	Bacteroidetes	Prevotella sp.	526
S108	Firmicutes	Lachnospiraceae [G-3] sp.	100
S412	Firmicutes	Enterococcus saccharolyticus	802
S189	Firmicutes	Peptostreptococcaceae [XI][G-4] sp.	369
**S149**	Firmicutes	Lactobacillus vaginalis	51
S387^*e*^	Firmicutes	Enterococcus durans	880
**S241**	Fusobacteria	Leptotrichia sp.	218
S203	Fusobacteria	Fusobacterium sp.	370
**S122**	Fusobacteria	Leptotrichiaceae [G-1] sp.	210
**S128**	Proteobacteria	Haemophilus parainfluenzae	718
S71	Spirochaetes	Treponema sp.	235
S200	Spirochaetes	Treponema sp.	226
Iron			
**S408**	Actinobacteria	Bifidobacterium dentium	588
**S291**	Actinobacteria	Olsenella sp.	807
**S69**	Actinobacteria	Corynebacterium matruchotii	666
**S295**	Actinobacteria	Actinomyces sp.	897
S276	Bacteroidetes	Porphyromonas sp.	275
**S68**	Bacteroidetes	Flavobacteriales [G-1] sp.	318
S40	Bacteroidetes	Capnocytophaga sp.	332
S36	Bacteroidetes	Prevotella denticola	291
**S260**	Firmicutes	Enterococcus italicus	803
S198	Firmicutes	Peptostreptococcaceae [XI][G-5] sp.	493
**S375**	Proteobacteria	Ralstonia sp.	27
S1	Proteobacteria	Neisseria subflava	476

a No. indicates the number of the species in the RDA plot (see Fig. S4); boldface font indicates a correlation of >0.996 (±5°).

b HOT indicates the human oral taxon ID in the Human Oral Microbiome Database (http://www.homd.org/index.php).

c Non-caries-associated species.

d Caries-associated species.

e Low-abundance species with a community-wide impact.

f Correlation value of >0.985 (±10°) (Fig. 5b).

Moreover, at the species level, the salivary pH, iron concentration, and DMFT index also exhibited substantial effects. For example, Ochrobactrum anthropi oral taxon 544 and TM7 [G-4] sp. strain oral taxon 355 showed strong negative and positive correlations, respectively, with the DMFT index. Alternatively, Prevotella multisaccharivorax oral taxon 794 and Enterococcus italicus oral taxon 803 exhibited strong negative correlations with the salivary pH and iron concentration, respectively. In addition, Lactobacillus salivarius oral taxon 756 and Leptotrichia sp. strain oral taxon 847 overlapped with the caries-associated species based on differential distribution analysis.

## DISCUSSION

In the present study, we examined the similarities and differences in salivary bacterial communities between individuals with and without caries using high-throughput sequencing. Because it is relatively stable, easy, and inexpensive to acquire, saliva is an almost ideal biological secretion for studies of the oral microbial profile in health and disease (18, 19). However, it also should be noted that saliva might not completely represent the bacterial diversity at the disease site, which should be addressed in further studies and clinical applications (7, 20). To obtain a more comprehensive understanding, we included recent reports on caries-associated microbial populations (see Table S2 in the supplemental material). Eighty percent of these reports concerned children (0.6 to 8 years), early young adults (8 to 16 years), or young adults (18 to 22 years). Neither the bacterial diversity nor the caries-associated taxa identified have been consistent among studies, which may be related to differences in sampling and/or analysis methodologies, as well as differences in environmental factors (9). Therefore, further analyses at different sites are necessary to verify the identities of the critical taxa and to confirm whether these taxa are consistent from one population to the next. Furthermore, we should also focus on the entire microbial community structure in addition to the characteristic members (21). In our study, the microbiomes from 43 adults were investigated, and different distribution patterns were observed in the subjects with and without caries at the genus level. We previously reported similar results based on a higher Schoener's cooccurrence index in the caries group than in the healthy group (7). Simultaneously, given the higher frequencies of Schoener's index in the subjects with caries observed in the present study, we propose that bacterial assemblages exhibit an aggregation structure (i.e., high cooccurrence) more often in caries, suggesting that mutualistic or syntrophic and competitive interactions dominate (22, 23) in the caries and healthy groups, respectively. Of course, further experimental evidence is needed.

Furthermore, pairs with higher cooccurrence probabilities were profiled at both the genus and species level. However, the distribution patterns at the species level were similar in the caries and healthy groups. Different distribution patterns at different taxonomic levels were also previously reported in the oral cavity of healthy individuals (24), and we believe that the interactions of species are complex and that a deeper investigation of microorganisms at the species level is required.

### Relationships of the bacterial profiles with pH and iron concentration.

As expected, the structure of the salivary bacterial community was significantly influenced by the pH and iron concentration (*P* < 0.01). However, in the RDA plots at both the genus and species levels, obtuse angles between the pH and iron concentration were observed, suggesting opposite trends of the two factors. Most of the non-caries- and caries-associated taxa were strongly correlated with these variables, suggesting an important relationship between these characteristic taxa and the environment. Low pH has been proposed to cause a shift in acid-tolerant and acid-producing bacterial consortia, such as those observed in our study, namely, Lactobacillus vaginalis and Streptococcus mitis (25, 26), which favors the formation of caries lesions (11, 27). Additionally, in the current study, species strongly correlated with high iron included caries-associated bacteria such as Propionibacterium acidifaciens (28), acid-tolerant strains such as Streptococcus constellatus (26) and Prevotella oulorum (11), as well as Streptococcus oligofermentans, which has been reported to inhibit the overgrowth of cariogenic pathogens (29). Therefore, we hypothesize that both a low pH and high iron concentration influence salivary species, which further increases the risk of caries.

However, higher pH and lower iron may not necessarily be safe for oral health. In our pH-positive (higher pH) profiles, Streptococcus sobrinus, previously reported as an etiologic agent of dental caries (30), was also observed, although with a low relative abundance (average of approximately 0.001%) and low prevalence (<20% in total). Similarly, the lower iron profile also included the potentially caries-associated pathogenic species Bifidobacterium dentium (31), although with low relative abundance (average of approximately 0.001%) and low prevalence (<20% in total). In addition, higher pH and lower iron profiles harbored several periodontitis-assorted species, including Aggregatibacter actinomycetemcomitans, Dialister pneumosintes (32), Prevotella oralis (33), Capnocytophaga sputigena, Cardiobacterium hominis (34), and Treponema lecithinolyticum (35). All of these data suggest that either higher or lower pH or iron creates potentially pathogenic conditions, and thus a single product for daily oral care probably should be avoided; the ranges of pH and iron concentration necessary for maintaining a healthy oral environment require further investigation.

### Relationships among the DMFT index, pH, and iron concentration based on correlations with salivary microbiomes.

The DMFT index, an index of the dental caries burden, was also significantly correlated with the bacterial community structure (*P* < 0.01). In the RDA plot (Fig. 5), the clustered caries-associated genera exhibited closer relationships with the DMFT index than with the pH or iron concentration, indicating that their cooccurrence is more strongly influenced by the DMFT index. Additionally, a higher DMFT index was consistently observed along with a reduced pH and increased iron concentration (Fig. 5 and Fig. S4). These findings indicate a potential clinical method for assessment of lesion acidity based on intraoral pH measurement (12); however, they contradict the cariostatic properties of iron (15). It is well accepted that the development of dental caries involves the dissolution of the tooth structure by acid produced by oral bacteria as a result of the fermentation of dietary carbohydrates, and aciduric or acid-producing species are the major cariogenic species (36). A certain amount of iron could reduce enamel demineralization (15, 37–41) through the formation of an acid-resistant coating on the enamel surface (42). Although the studies mentioned above evaluated the effects of iron(II) supplements (often ferrous sulfate) coupled with sugar or acidic solutions, neither the actual iron concentration nor the pH in the oral cavity has been reported. In the present study, the iron concentration representing the entire iron element in saliva was detected, including iron(II) and iron(III). Iron(III) complex addition has been reported to have no cariostatic effect (43). Therefore, different valences of iron should be considered in the future. Additionally, studies based on *in vitro* experiments have reported that iron compounds can form on the enamel surface, which indicates that it is also essential to develop methods to increase the percentage of change in superficial hardness and prevent remineralization in addition to other adverse effects, such as toxicity and tooth staining (15, 38). The reduced capacity of artificial saliva to promote remineralization was more substantial under higher iron concentrations (15). In our study, the iron concentration showed strong relationships with several of the caries-associated species mentioned above, which may have disrupted the balance of the bacterial community structure. Given the significant correlation of the iron concentration with the pH, which was not observed with the DMFT index, we suspect that the data for the pH and iron concentration represent the real-time conditions of the collected samples, whereas the DMFT index represents activities that mainly occurred in the past. In addition, a lower pH could enhance the solubility of iron and increase the availability of iron for oral microbial growth (44), which might result in a microbiota with a potential clinical impact on the formation of caries (16). Therefore, a possible explanation for the lack of a significant correlation between the iron concentration and the DMFT index is that the low solubility of iron (16) under aqueous and neutral pH conditions (45) limited the consistent increase in the iron concentration along with the DMFT index. Alternatively, individuals who develop caries may harbor a higher prevalence of certain acid-producing or acid-tolerant species that prefer higher salivary iron concentrations, which is consistent with the higher iron profile described above. Considering the importance and significant influences of iron and pH on the salivary microbial community in the present and previous studies (16, 19), the pH-iron correlation might have, at least in part, contributed to the oral microorganisms. Consequently, we propose that long-term investigation of the conversion of the oral microbiota and its correlation with pH in the presence of iron should be conducted before iron-containing products can be recommended.

## MATERIALS AND METHODS

### Subjects and specimen collection.

Volunteers were selected from Meipo Village, Jishishan Autonomous County, Gansu Province, China, in September 2014. Individuals were excluded from this study for the following reasons: they had taken antibiotics during the previous 3 months or had received systemic periodontal treatment in the preceding year; they had a systemic disease or immune suppression; and/or they were pregnant or smokers. All subjects did not eat and had undergone a 12-h period without oral care prior to initiation of the study. A total of 21 subjects with inactive caries and 22 healthy subjects (24 to 56 years old) were recruited and classified into C and H groups (see Table S1 in the supplemental material), respectively. As this study involved experiments on humans, the study protocols conformed to the guidelines of the Declaration of Helsinki. After obtaining informed written consent and with approval of the Ethics Committee of Northwest University for Nationalities, 1 ml of unstimulated saliva was collected from each subject the next morning.

### pH and iron concentration measurements and DNA extraction.

One milliliter of unstimulated saliva was mixed with 20 ml normal saline for use in further experiments. All saline-saliva samples were centrifuged at 12,000 rpm for 5 min. Ten milliliters of supernatant was used for measurement of the pH with a calibrated FE20 FiveEasy Plus pH meter (Mettler-Toledo, Schwerzenbach, Switzerland). Two milliliters of supernatant was used for measurement of the iron concentration with a Thermo elemental atomic absorption spectrometer (Thermo Scientific, East Lyme, CT). Briefly, standards containing known concentrations of iron were prepared by serial dilutions. The standards and samples then were dried at 80°C and 130°C for 20 s and 10 s, respectively, ashed, and atomized at 1,200°C and 2,100°C for 10 s and 5 s, respectively. Spectrophotometric measurements of the samples were performed at a wavelength of 243.8 nm. A calibration curve was generated using the measurements for the standards and was then used to quantify the iron levels in the samples and blank (normal saline only); the difference between the sample and blank was the final value. The samples used for measurement of the pH and iron concentration were analyzed in triplicate. The entire pellet was used to extract salivary DNA, and salivary DNA extraction was performed using a Qiagen Stool minikit (Qiagen, Valencia, CA) as previously described (7).

### Highly parallel DNA sequencing.

The 16S rRNA gene V4-V5 region was amplified using the following primers: 5′-GTGCCAGCMGCCGCGG-3′ and 5′-CCGTCAATTCMTTTRAGTTT-3′. PCR amplification was performed with an Eppendorf Mastercycler EP gradient thermal cycler (Eppendorf, Hauppauge, NY) in a total volume of 25 μl containing 9 μl of sterilized water, 5 μl of 5× PCR GC high enhancer, 5 μl of 5× PCR buffer, 2 μl of 2.5 mM deoxynucleoside triphosphates (dNTPs), 2 μl of 200 ng/μl template DNA, 0.25 μl of 5 U/μl TaKaRa polymerase, and 1 μl of each primer (10 μM). The thermal cycling conditions were initial denaturation at 98°C for 5 min, followed by 27 cycles at 98°C for 30 s, 50°C for 30 s, and 72°C for 30 s, with a final extension at 72°C for 5 min. The PCR products (3 μl) were detected on an agarose gel (2.0%). Each PCR product was tagged with an index sequence at the 5′ end of the forward primer. Purified PCR amplicons were used to construct paired-end DNA libraries, which were then run on the Illumina MiSeq (250-bp paired-end reads) platform.

Quantitative Insights Into Microbial Ecology (QIIME) toolkit v.1.7.0 was used to trim the raw sequences (46). Reads that were shorter than 150 bp, those that contained any ambiguous bases, or those that contained a homopolymer of longer than 8 bp were removed, and chimeric sequences were identified and removed using the UCHIME tool of the mothur software package (v.1.31.2) (47, 48). All of the trimmed sequences were normalized to the same sequencing depth using mothur. The operational taxonomic units (OTUs) were clustered at 97% similarity using the uclust tool of QIIME software. Representative sequences for each OTU were searched against the Human Oral Microbiome Database (HOMD; http://www.homd.org/) (49).

### Statistical analysis.

The Shannon, Simpson, Chao, and ACE indices (50, 51) were calculated for α-diversity measurement using mothur. Weighted UniFrac distance matrices (52) were calculated using QIIME. To estimate β-diversity, principal coordinate analysis (PCoA) was performed using the R package vegan. The linear discriminant analysis effect size (LEfSe) method (53–55) was used to compare the bacterial community structures between the samples from the patients with and without caries, as in previous reports (7). Schoener's index (abundance-based) (56) was computed to measure the cooccurrence probability for each pair of genera or species using the R spaa package, which was then used to construct heatmaps to evaluate the different distribution patterns in the two groups using the ggplots package in R, as described previously (7). Redundancy analysis (RDA) was performed using the vegan package in R to test the influences of the salivary pH, iron concentration, and DMFT index on the microbial community structure at the species level. In the RDA plot of species, the arrows pointing to the factors were rotated 5 and 10 degrees positively and negatively, respectively. Species that fell in this interval were considered to have strong correlations (cos5° ≈ 0.996 and cos10° ≈ 0.985). In addition, the distance to the origin was calculated, and only values of >0.5 are shown in Table 1.

## Supplementary Material

Supplemental material

## References

[1] Anusavice KJ 2002 Dental caries: risk assessment and treatment solutions for an elderly population. Compend Contin Educ Dent 23:12–20. 12790012

[2] Petersen PE 2003 The World Oral Health Report 2003: continuous improvement of oral health in the 21st century–the approach of the WHO Global Oral Health Programme. Community Dent Oral Epidemiol 31(Suppl 1):S3–S23. 10.1046/j..2003.com122.x 15015736

[3] Cummins D 2010 Dental caries: a disease which remains a public health concern in the 21st century–the exploration of a breakthrough technology for caries prevention. J Clin Dent 21:25–37. 24156135

[4] Human Microbiome Project Consortium. 2012 Structure, function and diversity of the healthy human microbiome. Nature 486:207–214. doi:10.1038/nature11234. 22699609 PMC3564958

[5] Jiang W, Ling ZX, Lin XL, Chen YD, Zhang J, Yu JJ, Xiang C, Chen H 2014 Pyrosequencing analysis of oral microbiota shifting in various caries states in childhood. Microb Ecol 67:962–969. doi:10.1007/s00248-014-0372-y. 24504329

[6] Yang F, Zeng XW, Ning K, Liu KL, Lo CC, Wang W, Chen J, Wang DM, Huang RR, Chang XZ, Chain PS, Xie G, Ling JQ, Xu J 2012 Saliva microbiomes distinguish caries-active from healthy human populations. ISME J 6:1–10. doi:10.1038/ismej.2011.71. 21716312 PMC3246229

[7] Zhou J, Jiang N, Wang S, Hu X, Jiao K, He X, Li Z, Wang J 2016 Exploration of human salivary microbiomes–insights into the novel characteristics of microbial community structure in caries and caries-free subjects. PLoS One 11:e0147039. doi:10.1371/journal.pone.0147039. 26784334 PMC4718657

[8] Sanchez MC, Llama-Palacios A, Blanc V, Leon R, Herrera D, Sanz M 2011 Structure, viability and bacterial kinetics of an in vitro biofilm model using six bacteria from the subgingival microbiota. J Periodont Res 46:252–260. doi:10.1111/j.1600-0765.2010.01341.x. 21261622

[9] Wang X, Willing MC, Marazita ML, Wendell S, Warren JJ, Broffitt B, Smith B, Busch T, Lidral AC, Levy SM 2012 Genetic and environmental factors associated with dental caries in children: the Iowa Fluoride Study. Caries Res 46:177–184. doi:10.1159/000337282. 22508493 PMC3580152

[10] Marsh PD 2009 Dental plaque as a biofilm: the significance of pH in health and caries. Compend Contin Educ Dent 30:76–90. 19301526

[11] Kianoush N, Adler CJ, Nguyen KA, Browne GV, Simonian M, Hunter N 2014 Bacterial profile of dentine caries and the impact of pH on bacterial population diversity. PLoS One 9:e92940. doi:10.1371/journal.pone.0092940. PMC3968045 24675997

[12] Kuribayashi M, Kitasako Y, Matin K, Sadr A, Shida K, Tagami J 2012 Intraoral pH measurement of carious lesions with qPCR of cariogenic bacteria to differentiate caries activity. J Dent 40:222–228. doi:10.1016/j.jdent.2011.12.013. 22222970

[13] Jalasvuori H, Haukioja A, Tenovuo J 2012 Probiotic Lactobacillus reuteri strains ATCC PTA 5289 and ATCC 55730 differ in their cariogenic properties in vitro. Arch Oral Biol 57:1633–1638. doi:10.1016/j.archoralbio.2012.07.014. 23010217

[14] Ravel J, Gajer P, Abdo Z, Schneider GM, Koenig SS, McCulle SL, Karlebach S, Gorle R, Russell J, Tacket CO, Brotman RM, Davis CC, Ault K, Peralta L, Forney LJ 2011 Vaginal microbiome of reproductive-age women. Proc Natl Acad Sci U S A 108(Suppl 1):S4680–S4687. 10.1073/pnas.1002611107 PMC3063603 20534435

[15] Alves KM, Franco KS, Sassaki KT, Buzalaf MA, Delbem AC 2011 Effect of iron on enamel demineralization and remineralization in vitro. Arch Oral Biol 56:1192–1198. doi:10.1016/j.archoralbio.2011.04.011. 21555115

[16] Wang R, Kaplan A, Guo L, Shi W, Zhou X, Lux R, He X 2012 The influence of iron availability on human salivary microbial community composition. Microb Ecol 64:152–161. doi:10.1007/s00248-012-0013-2. 22318873 PMC3376180

[17] Seow WK 2012 Environmental, maternal, and child factors which contribute to early childhood caries: a unifying conceptual model. Int J Paediatr Dent 22:157–168. doi:10.1111/j.1365-263X.2011.01186.x. 21972925

[18] Baum BJ, Yates JR III, Srivastava S, Wong DT, Melvin JE 2011 Scientific frontiers: emerging technologies for salivary diagnostics. Adv Dent Res 23:360–368. doi:10.1177/0022034511420433. 21917746 PMC3172997

[19] Giannobile WV, McDevitt JT, Niedbala RS, Malamud D 2011 Translational and clinical applications of salivary diagnostics. Adv Dent Res 23:375–380. doi:10.1177/0022034511420434. 21917748 PMC3172998

[20] Simon-Soro A, Mira A 2015 Solving the etiology of dental caries. Trends Microbiol 23:76–82. doi:10.1016/j.tim.2014.10.010. 25435135

[21] Abusleme L, Dupuy AK, Dutzan N, Silva N, Burleson JA, Strausbaugh LD, Gamonal J, Diaz PI 2013 The subgingival microbiome in health and periodontitis and its relationship with community biomass and inflammation. ISME J 7:1016–1025. doi:10.1038/ismej.2012.174. 23303375 PMC3635234

[22] Gotelli NJ, McCabe DJ 2002 Species co-occurrence: a meta-analysis of J. M. Diamond's assembly rules model. Ecology 83:2091–2096.

[23] Horner-Devine MC, Silver JM, Leibold MA, Bohannan BJM, Colwell RK, Fuhrman JA, Green JL, Kuske CR, Martiny JBH, Muyzer G, Ovreas L, Reysenbach AL, Smith VH 2007 A comparison of taxon co-occurrence patterns for macro- and microorganisms. Ecology 88:1345–1353. doi:10.1890/06-0286. 17601127

[24] Bik EM, Long CD, Armitage GC, Loomer P, Emerson J, Mongodin EF, Nelson KE, Gill SR, Fraser-Liggett CM, Relman DA 2010 Bacterial diversity in the oral cavity of 10 healthy individuals. ISME J 4:962–974. doi:10.1038/ismej.2010.30. 20336157 PMC2941673

[25] Boskey ER, Telsch KM, Whaley KJ, Moench TR, Cone RA 1999 Acid production by vaginal flora in vitro is consistent with the rate and extent of vaginal acidification. Infect Immun 67:5170–5175. 10496892 10.1128/iai.67.10.5170-5175.1999 PMC96867

[26] Svensater G, Borgstrom M, Bowden GHW, Edwardsson S 2003 The acid-tolerant microbiota associated with plaque from initial caries and healthy tooth surfaces. Caries Res 37:395–403. doi:10.1159/000073390. 14571116

[27] Takahashi N, Nyvad B 2011 The role of bacteria in the caries process: ecological perspectives. J Dent Res 90:294–303. doi:10.1177/0022034510379602. 20924061

[28] Wolff D, Frese C, Maier-Kraus T, Krueger T, Wolff B 2013 Bacterial biofilm composition in caries and caries-free subjects. Caries Res 47:69–77. doi:10.1159/000344022. 23147531

[29] Liu L, Tong HC, Dong XZ 2012 Function of the pyruvate oxidase-lactate oxidase cascade in interspecies competition between Streptococcus oligofermentans and Streptococcus mutans. Appl Environ Microbiol 78:2120–2127. doi:10.1128/AEM.07539-11. 22287002 PMC3302633

[30] Oda Y, Hayashi F, Okada M 2015 Longitudinal study of dental caries incidence associated with Streptococcus mutans and Streptococcus sobrinus in patients with intellectual disabilities. BMC Oral Health 15:102. doi:10.1186/s12903-015-0087-6. 26328921 PMC4557917

[31] Modesto M, Biavati B, Mattarelli P 2006 Occurrence of the family Bifidobacteriaceae in human dental caries and plaque. Caries Res 40:271–276. doi:10.1159/000092237. 16707878

[32] Louhelainen AM, Aho J, Tuomisto S, Aittoniemi J, Vuento R, Karhunen PJ, Pessi T 2014 Oral bacterial DNA findings in pericardial fluid. J Oral Microbiol 6:25835. 25412607 10.3402/jom.v6.25835 PMC4239404

[33] Nadkarni MA, Browne GV, Chhour KL, Byun R, Nguyen KA, Chapple CC, Jacques NA, Hunter N 2012 Pattern of distribution of Prevotella species/phylotypes associated with healthy gingiva and periodontal disease. Eur J Clin Microbiol 31:2989–2999. doi:10.1007/s10096-012-1651-5. 22684253

[34] Colombo APV, Bennet S, Cotton SL, Goodson JM, Kent R, Haffajee AD, Socransky SS, Hasturk H, Van Dyke TE, Dewhirst FE, Paster BJ 2012 Impact of periodontal therapy on the subgingival microbiota of severe periodontitis: comparison between good responders and individuals with refractory periodontitis using the human oral microbe identification microarray. J Periodontol 83:1279–1287. doi:10.1902/jop.2012.110566. 22324467 PMC3971922

[35] Riep B, Edesi-Neuss L, Claessen F, Skarabis H, Ehmke B, Flemmig TF, Bernimoulin JP, Gobel UB, Moter A 2009 Are putative periodontal pathogens reliable diagnostic markers? J Clin Microbiol 47:1705–1711. doi:10.1128/JCM.01387-08. 19386852 PMC2691128

[36] Duran-Pinedo AE, Frias-Lopez J 2015 Beyond microbial community composition: functional activities of the oral microbiome in health and disease. Microbes Infect 17:505–516. doi:10.1016/j.micinf.2015.03.014. 25862077 PMC4495649

[37] Buzalaf MAR, Italiani FD, Kato MT, Martinhon CCR, Magalhaes AC 2006 Effect of iron on inhibition of acid demineralisation of bovine dental enamel in vitro. Arch Oral Biol 51:844–848. doi:10.1016/j.archoralbio.2006.04.007. 16782041

[38] Kato MT, Sales-Peres SH, Buzalaf MA 2007 Effect of iron on acid demineralisation of bovine enamel blocks by a soft drink. Arch Oral Biol 52:1109–1111. doi:10.1016/j.archoralbio.2007.04.012. 17559795

[39] Martinhon CCR, de Moraes Italiani FD, de Magalhaes Padilha P, Bijella MFTB, Delbem ACB, Buzalaf MAR 2006 Effect of iron on bovine enamel and on the composition of the dental biofilm formed “in situ.” Arch Oral Biol 51:471–475. 16307723 10.1016/j.archoralbio.2005.10.003

[40] Pecharki GD, Cury JA, Leme AFP, Tabchoury CPM, Cury AAD, Rosalen PL, Bowne WH 2005 Effect of sucrose containing iron (II) on dental biofilm and enamel demineralization in situ. Caries Res 39:123–129. doi:10.1159/000083157. 15741724

[41] Sales-Peres SHC, Pessan JP, Buzalaf MAR 2007 Effect of an iron mouthrinse on enamel and dentine erosion subjected or not to abrasion: an in situ/ex vivo study. Arch Oral Biol 52:128–132. doi:10.1016/j.archoralbio.2006.08.010. 17045952

[42] Torell P 1988 Iron and dental caries. Swed Dent J 12:113–124. 3165568

[43] Al-Shalan TA 2009 In vitro cariostatic effects of various iron supplements on the initiation of dental caries. Saudi Dent J 21:117–122. doi:10.1016/j.sdentj.2009.05.001. 23960469 PMC3723110

[44] Andrews SC, Robinson AK, Rodriguez-Quinones F 2003 Bacterial iron homeostasis. FEMS Microbiol Rev 27:215–237. doi:10.1016/S0168-6445(03)00055-X. 12829269

[45] Schaible UE, Kaufmann SH 2004 Iron and microbial infection. Nat Rev Microbiol 2:946–953. doi:10.1038/nrmicro1046. 15550940

[46] Caporaso JG, Kuczynski J, Stombaugh J, Bittinger K, Bushman FD, Costello EK, Fierer N, Pena AG, Goodrich JK, Gordon JI, Huttley GA, Kelley ST, Knights D, Koenig JE, Ley RE, Lozupone CA, McDonald D, Muegge BD, Pirrung M, Reeder J, Sevinsky JR, Turnbaugh PJ, Walters WA, Widmann J, Yatsunenko T, Zaneveld J, Knight R 2010 QIIME allows analysis of high-throughput community sequencing data. Nat Methods 7:335–336. doi:10.1038/nmeth.f.303. 20383131 PMC3156573

[47] Edgar RC, Haas BJ, Clemente JC, Quince C, Knight R 2011 UCHIME improves sensitivity and speed of chimera detection. Bioinformatics 27:2194–2200. doi:10.1093/bioinformatics/btr381. 21700674 PMC3150044

[48] Schloss PD, Westcott SL, Ryabin T, Hall JR, Hartmann M, Hollister EB, Lesniewski RA, Oakley BB, Parks DH, Robinson CJ, Sahl JW, Stres B, Thallinger GG, Van Horn DJ, Weber CF 2009 Introducing mothur: open-source, platform-independent, community-supported software for describing and comparing microbial communities. Appl Environ Microbiol 75:7537–7541. doi:10.1128/AEM.01541-09. 19801464 PMC2786419

[49] Chen T, Yu WH, Izard J, Baranova OV, Lakshmanan A, Dewhirst FE 2010 The Human Oral Microbiome Database: a web accessible resource for investigating oral microbe taxonomic and genomic information. Database 2010:baq013. 20624719 10.1093/database/baq013 PMC2911848

[50] Chao A, Lee SM, Jeng SL 1992 Estimating population-size for capture recapture data when capture probabilities vary by time and individual animal. Biometrics 48:201–216. doi:10.2307/2532750. 1581485

[51] Chao A, Shen TJ 2003 Nonparametric estimation of Shannon's index of diversity when there are unseen species in sample. Environ Ecol Stat 10:429–443. doi:10.1023/A:1026096204727.

[52] Lozupone C, Knight R 2005 UniFrac: a new phylogenetic method for comparing microbial communities. Appl Environ Microbiol 71:8228–8235. doi:10.1128/AEM.71.12.8228-8235.2005. 16332807 PMC1317376

[53] Blankenberg D, Von Kuster G, Coraor N, Ananda G, Lazarus R, Mangan M, Nekrutenko A, Taylor J 2010 Galaxy: a web-based genome analysis tool for experimentalists. Curr Protoc Mol Biol Chapter 19:Unit 19.10.1-21. doi:10.1002/0471142727.mb1910s89. PMC4264107 20069535

[54] Giardine B, Riemer C, Hardison RC, Burhans R, Elnitski L, Shah P, Zhang Y, Blankenberg D, Albert I, Taylor J, Miller W, Kent WJ, Nekrutenko A 2005 Galaxy: a platform for interactive large-scale genome analysis. Genome Res 15:1451–1455. doi:10.1101/gr.4086505. 16169926 PMC1240089

[55] Goecks J, Nekrutenko A, Taylor J, Galaxy Team 2010 Galaxy: a comprehensive approach for supporting accessible, reproducible, and transparent computational research in the life sciences. Genome Biol 11:R86. doi:10.1186/gb-2010-11-8-r86. 20738864 PMC2945788

[56] Schoener TW 1992 Overdispersed niches on a crowded island—a Citation Classic commentary on the Anolis lizards of Bimini: resource partitioning in a complex fauna. Curr Contents Agric Biol Environ Sci 6:10–10.

