# Supplementary material for: Influences of pH and Iron Concentration on the Salivary Microbiome in Individual Humans with and without Caries
Source: Appl Environ Microbiol. 2017 Feb 1;83(4):e02412-16. doi: 10.1128/AEM.02412-16 (PMC5288818; doi:10.1128/AEM.02412-16)
Supplement: Supplemental material [file supp_83_4_e02412-16__index.html]

Supplemental material 

# Influences of pH and Iron Concentration on the Salivary Microbiome in Individual Humans with and without Caries

## Supplemental material

- Supplemental file 1 -

  Principal-coordinate analysis-based estimations of β-diversity variation in saliva of individuals with or without caries (Fig. S1); bacterial taxonomic comparisons of saliva samples from individuals with or without caries (Fig. S2); distribution patterns at the species level (Fig. S3); redundancy analysis of the significant influences of the salivary pH, iron concentration, and DMFT index on the bacterial community structure at the species level (Fig. S4); characteristics of individual participants in the study (Table S1); summary of studies on bacterial communities in samples from individuals with or without caries (Table S2); taxonomic assignment of abbreviations in Fig. S4 (Table S3).

  PDF, 1.4M
