# Supplementary material for: Influences of pH and Iron Concentration on the Salivary Microbiome in Individual Humans with and without Caries
Source: Appl Environ Microbiol. 2017 Feb 1;83(4):e02412-16. doi: 10.1128/AEM.02412-16 (PMC5288818; doi:10.1128/AEM.02412-16)
Supplement: Supplemental material [file AEM.02412-16_zam999117663s1.pdf]

Influences of pH and iron on the salivary microbiome in individuals with and without caries

Running title: Influences of pH and Fe on microbes in caries

Jianye Zhou[a]*, Nan Jiang[b]*, Zhengzheng Wang[c], Longqing Li[d], Jumei Zhang[a], Rui Ma[c], Hongbing Nie[a], Zhiqiang Li[a #].

Key Laboratory of Oral Diseases of Gansu Province, Key Laboratory of Stomatology of State Ethnic Affairs Commission, Northwest University for Nationalities, Lanzhou 730030, Gansu, China[a]; Institute of Applied Ecology, Chinese Academy of Sciences, Shenyang, China[b]; School of Stomatology, Lanzhou University, Lanzhou, China[c]; Institute of Modern Physics, Chinese Academy of Sciences, Lanzhou, China[d]

[#] Address correspondence to Zhiqiang Li,

Tel.: +86 13519607866

E-mail: sciuse1@163.com

* Jianye Zhou and Nan Jiang contributed equally to this paper.

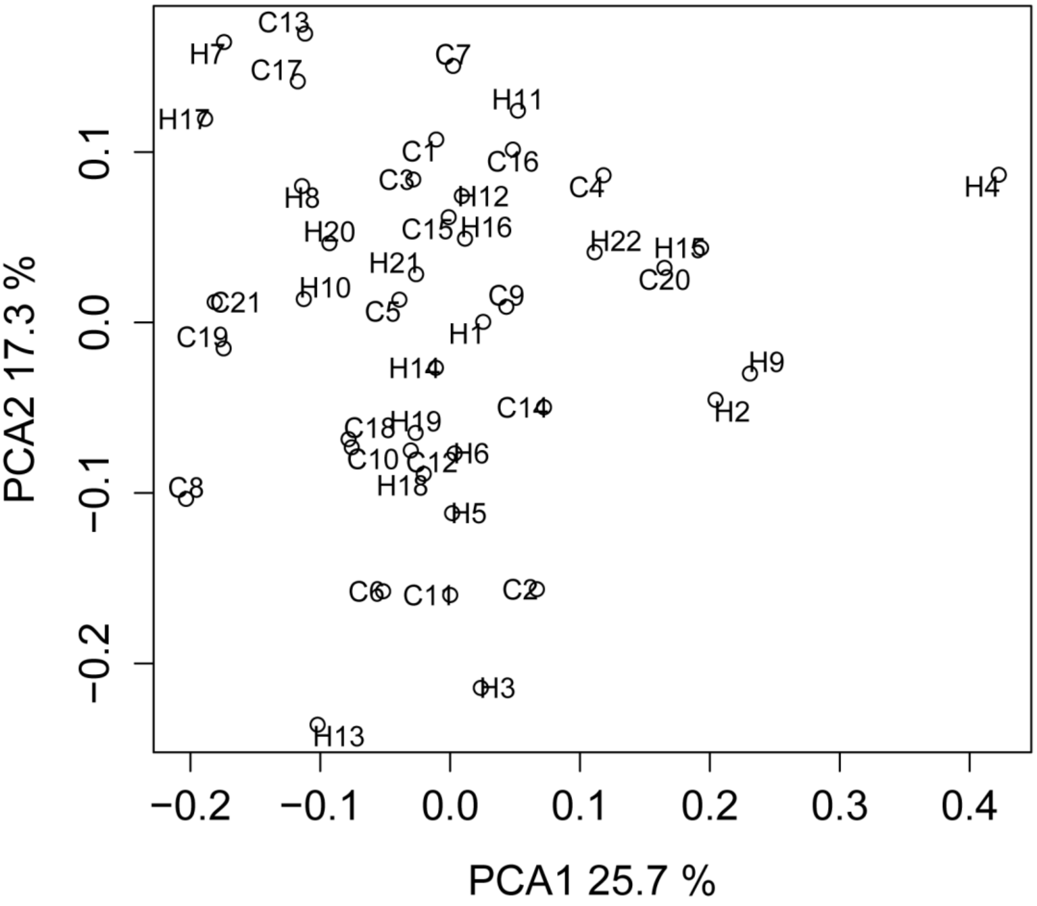

Fig. S1 Principal coordinate analysis (PCoA) was used to estimate the β-diversity variation between saliva from individuals with (C) and without (H) caries using a UniFrac phylogenetic distance matrix.

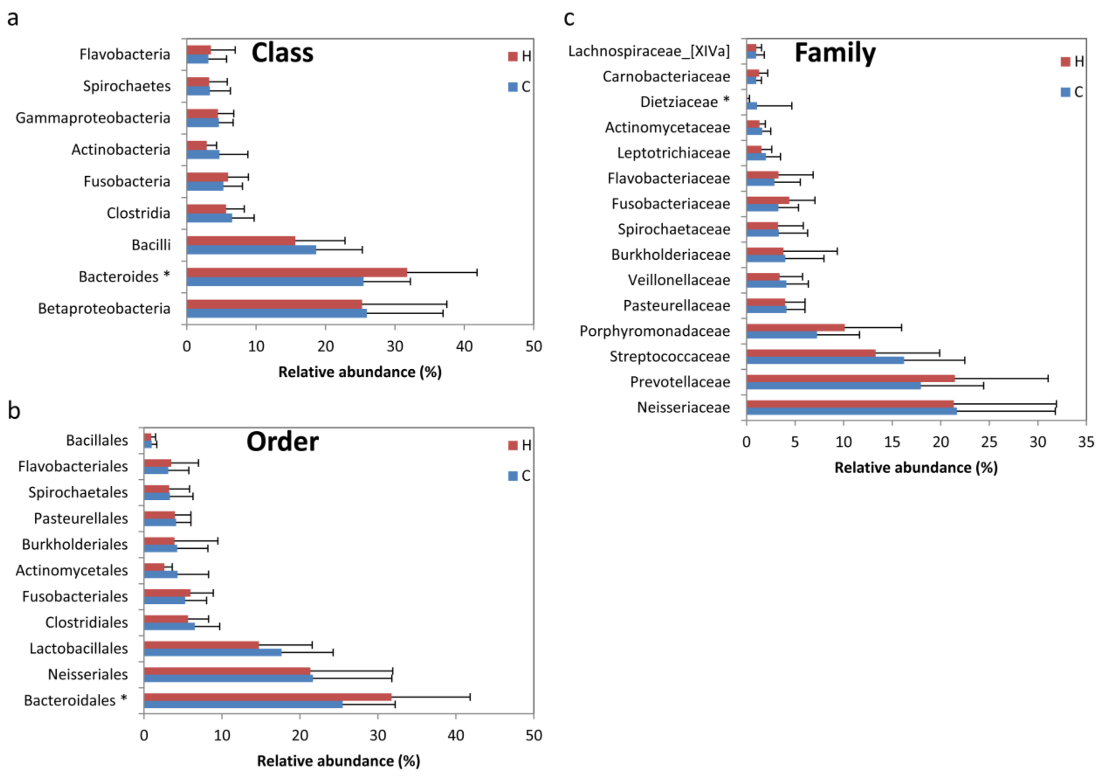

Fig. S2 Comparisons of bacterial taxonomy (>1% of relative abundance) of samples from individuals with (C) and without (H) caries at the class (a), order (b), and family (c) levels. * P<0.05.

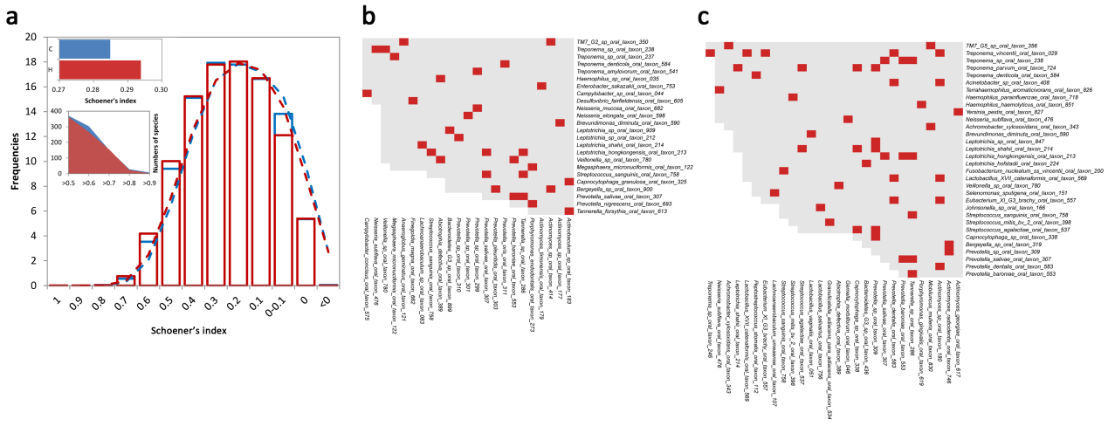

Fig. S3 Distribution patterns at the species level. (a) The frequency for each interval of Schoener's index was calculated in the caries and healthy groups, respectively. The numbers of involved genera at different intervals and global comparison of schoener's index between the two groups (by T-test) were also showed within the plot. All pairs with higher schoener's index (>0.8) were showed in the healthy (b) and caries (c) groups.

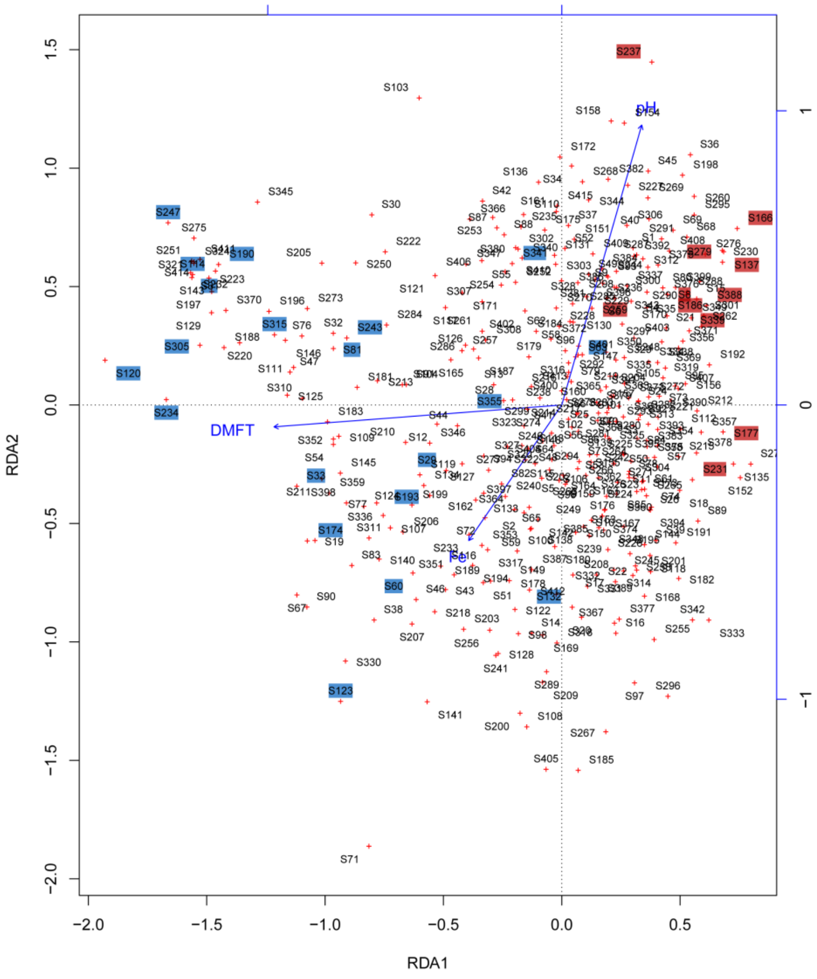

Fig. S4 Redundancy analysis (RDA) of the significant influences of the salivary pH, iron concentration, and DMFT index on the bacterial community structure at the species level. Red and blue indicate the health- and caries-associated taxa, respectively. The taxonomic information is detailed in Table S3.

Table S1 Characteristics of individuals in the study

Group	Age	N (Female)	DMFT
Caries (C)	40.4±6.5	7 (33.3%)	4.1±3.4***
Health (H)	38.7±9.0	12 (54.5%)	0

*** indicates that difference is significant at the 0.001 level.

Table S2 Summary of studies on bacterial community from samples with and without caries

Reference	Method	Sequences[#]	Age (yrs)	Scale (H:C)	Sample	Area	alpha-diversity	Caries-associated Genera	Health-associated Genera	Caries-associated Species	Health-associated Species
here	Miseq, V4-V5	13,749 rps	24-55	22:21	Saliva	Gansu, China	NS	In test, Figs. 1b and 2a	In test, Figs. 1b and 2a	In test, Figs. 1c and 2b	In test, Figs. 1c and 2b
(1)	Miseq, V4	12,638 rps	25-55	10:10	Saliva	Gansu, China	NS	*Veillonella*	*Neisseria*	NR	NR
								Bifidobacterium			
								Parascardovia			
								Chryseobacterium			
								Terrimonas			
								Burkholderia			
								Sporobacter			
								Selenomonas			
								Olsenella			
								Scardovia			
(2)	454-pyrosequencing, V3-V4	4900 rps	3	11:11	mucosa-adherent biofilm & dental plaque	Northern Sweden	H>C	NS		***Actinobaculum sp. HOT 183***	*Actinomyces sp. HOT 177*
										Atopobium parvulum	*Bergeyella sp. HOT 322*
										Aggregatibacter sp. HOT 513	*Campylobacter concisus*
										Streptococcus sp. HOT 431	*Granulicatella adiacens*
										Streptococcus oralis	*Kingella genus*
										S.mitis/Smitis bv2/ S. infantis	*Kingella dentifricans*
											Kingella oralis
											Leptrotrichia hofstadii
											Leptricihia sp. HOT 223 or 234
											Streptococcus

											anginosus/S.gordonii
											Streptococcus sanguinis
(3)	454-pyrosequencing, V3-V5	2724 rps	68.6-82.2	21:21	Supra-Gingival plaque	Wuhan, China	NS	*Atopobium*	*Aggregatibacter*	***Olsenella profusa***	*Bergeyella sp.*
								Megasphaera	*BacteroidetesG2*	***Oribacterium sp.***	*Catonella morbi*
								Prevotella	*Tannerella*	***Propionibacterium acidifaciens***	***Alloprevotella sp.***
								Veillonella	*Fusobacterium*	*Streptococcus mutans*	*Bacteroidetes[G-2] sp.*
								Streptococcus	*Porphyromonas*	*Prevotella multisaccharivorax*	***Propionibacterium propionicum***
								Actinomyces	*Corynebacterium*	***Lactobacillus crispatus***	*Lachnoanaerobaculum umeaense*
								Anaeroglobus	*Delftia*	*Prevotella oralis*	*Lachnoanaerobaculum sp.*
								Oribacterium	*Capnocytophaga*		*Cardiobacterium valvarum*
								Pseudoramibacter	*Cardiobacterium*		*Prevotella saccharolytica*
								Lactobacillus	*Catonella*		*Gemella sanguinis*
								Propionibacterium	*BacteroidetesG5*		*Fretibacterium sp.*
								Olsenella	*Gemella*		*Tannerella sp.*
									LachnospiraceaeG3		*Delfita acidovorans*
									ClostridialesF2G1		*Prevotella intermedia*
									Bergeyella		*Peptococcus sp.*
									Lachnoanaerobaculum		*Bacteriodetes[G-5] sp.*
(4)	454-pyrosequencing, V1-V3	4217 rps	13-24.5 ms*	9:10	dental plaque from intact enamel	Beijing, China	NS	NS	*Fusobacterium*	Streptococcus mutans	
									Capnocytophaga		
(5)	454-pyrosequencing, V1-V3	4805 rps	3-7	30:30	Supragingival plaques	Zhejiang, China	H>C	*Crptobacterium*	*Capnocytophaga*	NR	NR

								Lactobacillus	*Fusobacterium*		
								Megasphaera	*Porphyromonas*		
								Olsenella	*Abiotrophia*		
								Scardovia	*Comamonas*		
								Shuttleworthia	*Tennerella*		
								Cryptobacterium	*Eikenella*		
								Streptococcus	*Paludibacter*		
								Actinomyces	*Treponema*		
								Corynebacterium	*Actinobaculum*		
								Flavobacterium	*Stenotrophomonas*		
								Neisseria	*Aestuariimicrobium*		
								Bergeyella	*Peptococcus*		
								Derxia			
(6)	HOMIM	NR	47-56	174-447	Saliva	Eriksen et al	NR	NR	NR	*Streptococcus salivarius*	NR
										Solobacterium moorei	
										Streptococcus parasanguinisI and II and sp. clone BE024 ot057/411/721 *Streptococcus parasanguinis I and II and sinensis ot411/721/767,* *S. salivarius and sp. clone FO042 ot067/755*	,
(7)	DGGE, V3-V5	189 cia	8-32 ms*	12:12	Supragingival plaque	Guangzhou, Chian	H>C	NS			
(8)	HOMIM, Full length	NR	6-8	20:30	Saliva	Guizhou, China	C>H	*Leptotrichia*	NR	*Bacteroidetes[G-2] sp _ot274*	*Gemella haemolysans_ot626*
										Capnocytophaga sputigena_ot775	*Granulicatella elegans_ot596*

										Tannerella sp_ot286	*Streptococcus infantis and sp clone FN042_ot065_638*
										Campylobacter showae_ot763	*Streptococcus mitis bv2 and sp clone FP064_ot069_398*
										Campylobacter Cluster I_ot580_748_763	*Streptococcus sp clone F0042_ot067*
										Selenomonas infelix _ot126 and related species ot479_ot481_ ot639_ ot054	*Rothia dentocariosa_ot587*
										Parvimonas micra_ot111	
										Leptotrichia hofstadii_ot224	
(9)	454-pyrosequencing, V4-V5	9458 rps	18-22	26:19	Saliva	Guangzhou, China	NS	*Prevotella*	***Oribacterium***	*Prevotella histicola*	*Prevotella sp.*
									Porphyromonas		
(10)	Cultivable Bacterial-PCR-Sanger, V3 or V4	5608 cia	2-6	40:42	buccal and interproximal surfaces	Cambridge, USA	NR	NR	NR	*Veillonella parvula HOT 161*	*Leptotrichia hofstadii HOT 224*
										Actinomyces gerencseriae HOT 618	***Selenomonas dianae HOT 139***
										Streptococcus mutans HOT 686	*Capnocytophaga granulosa HOT 325*
										Scardovia wiggsiae HOT 195	*Leptotrichia sp. HOT 071*
										Porphyromonas catoniae HOT 283	*Streptococcus constellatus HOT 758*
										Parascardovia denticolens HOT 586	*Actinomyces sp. HOT 175*
										Streptococcus sobrinus HOT 768	*Actinomyces sp. HOT 169*
											Eubacterium saburreum(14)(G1)HOT 494
											Leptotrichia wadei HOT 222
											Actinomyces sp. HOT 177
											Actinomyces sp. HOT 180
											Streptococcus gordonii HOT 622

											Streptococcus mitis HOT 677
											Veillonella dispar HOT 160
											Streptococcus intermedius HOT 644
											Streptococcus thermophilus HOT C65
(11)	Sanger sequencing, V5-V9	5299 cia	7-16	18:20	dental plaque	Columbus, USA	H>C	***Lactobacillus***	*Streptococcus*	*Propionibacterium FMA5*	*Streptococcus pneumoniae-Streptococcus infantis*
								Propionibacterium	*Corynebacterium*		*Streptococcus gordonii*
									Lachnospiraceae		*Corynebacterium matruchotii*
									Gemella		*Streptococcus cristatus*
									Capnocytophaga		*Capnocytophaga gingivalis*
(12)	Sanger, V1-V3	3802 cia	2-6	41:39	plaque from cavities	Boston, USA	NR	NS	NS	*Granulicatella elegans*	*Capnocytophaga gingivalis*
										Veillonella sp. HOT-780	*Abiotrophia defectiva*
										Streptococcus mutans	*Lachnospiraceae sp. HOT-100*
										Bifidobacteriaceae spp.	*Streptococcus sanguinis*
											Streptococcus cristatus
(13)	DGGE & 454-pyrosequencing, V3	1557 rps	3-6	28:32	saliva and plaque	Hangzhou, China	NS	*Veillonella*	*Streptococcus*	NS	NS
								Actinomyces	*Granulicatella*		
								Leptotrichia	*Thiomonas*		
(14)	DGGE, V4	396 cia	2.4-8.6	8:12	buccogingival surfaces and accessible proximal surfaces	New York, USA	H>C	*Bacteroidetes*	*Leptotrichia*	NR	NR
								Fusobacterium	*Neisseria*		
								Filifactor	*Streptococcus*		

								Leptotrichia			
								Corynebacterium			
								Prevotella			
								Lautropia			
								Treponema			
(15)	Cultivable Bacterial-PCR-DGGE, V4	86 cia	18-22	9:11	Saliva	Birmingham, USA	H>C	NR	NR	*Streptococcus mutans*	NR

[#] rps and cia indicate reads per sample and clones in all, respectively. [*] ms indicates months. NR and NS indicate not-reported and not-significantly, respectively. Bold word represents the taxa that were the same with our study.

Table S3 Taxonomic assignment of abbreviations in Fig. S4

No	Phylum	Class	Order	Family	Genera	Species	No	Phylum	Class	Order	Family	Genera	Species
S1	Proteobacteria	Betaproteobacteria	Neisseriales	Neisseriaceae	*Neisseria*	*subflava oral taxon 476*	S209	Firmicutes	Clostridia	Clostridiales	Lachnospiraceae_[XIVa]	*Lachnospiraceae_[G-5]*	*sp. oral taxon 080*
S2	Firmicutes	Bacilli	Lactobacillales	Streptococcaceae	*Streptococcus*	*mitis bv 2 oral taxon 398*	S210	Proteobacteria	Betaproteobacteria	Neisseriales	Neisseriaceae	*Kingella*	*denitrificans oral taxon 582*
S3	Bacteroidetes	Bacteroides	Bacteroidales	Prevotellaceae	*Prevotella*	*melaninogenica oral taxon 469*	S211	TM7	TM7_[C-1]	TM7_[O-1]	TM7_[F-1]	*TM7_[G-3]*	*sp. oral taxon 351*
S4	Bacteroidetes	Bacteroides	Bacteroidales	Porphyromonadaceae	*Porphyromonas*	*sp. oral taxon 279*	S212	Proteobacteria	Gammaproteobacteria	Cardiobacteriales	Cardiobacteriaceae	*Cardiobacterium*	*valvulum oral taxon 540*
S5	Bacteroidetes	Bacteroides	Bacteroidales	Prevotellaceae	*Prevotella*	*intermedia oral taxon 643*	S213	Spirochaetes	Spirochaetes	Spirochaetales	Spirochaetaceae	*Treponema*	*sp. oral taxon 508*
S6	Bacteroidetes	Bacteroides	Bacteroidales	Porphyromonadaceae	*Porphyromonas*	*gingivalis oral taxon 619*	S214	Bacteroidetes	Bacteroides	Bacteroidales	Prevotellaceae	*Prevotella*	*sp. oral taxon 305*
S7	Proteobacteria	Betaproteobacteria	Burkholderiales	Burkholderiaceae	*Lautropia*	*mirabilis oral taxon 022*	S215	Fusobacteria	Fusobacteria	Fusobacteriales	Leptotrichiaceae	*Leptotrichia*	*sp. oral taxon 225*
S8	Fusobacteria	Fusobacteria	Fusobacteriales	Fusobacteriaceae	*Fusobacterium*	*periodonticum oral taxon 201*	S216	Proteobacteria	Betaproteobacteria	Neisseriales	Neisseriaceae	*Neisseria*	*sp. oral taxon 499*
S9	Proteobacteria	Gammaproteobacteria	Pasteurellales	Pasteurellaceae	*Terrahaemophilus*	*aromaticivorans oral taxon 826*	S217	Firmicutes	Clostridia	Clostridiales	Veillonellaceae	*Anaeroglobus*	*geminatus oral taxon 121*
S10	Firmicutes	Clostridia	Clostridiales	Veillonellaceae	*Veillonella*	*parvula oral taxon 161*	S218	Firmicutes	Clostridia	Clostridiales	Lachnospiraceae_[XIVa]	*Moryella*	*sp. oral taxon 419*
S11	Bacteroidetes	Bacteroides	Bacteroidales	Prevotellaceae	*Alloprevotella*	*sp. oral taxon 473*	S219	Firmicutes	Clostridia	Clostridiales	Lachnospiraceae_[XIVa]	*Lachnospiraceae_[G-2]*	*sp. oral taxon 096*
S12	Proteobacteria	Betaproteobacteria	Neisseriales	Neisseriaceae	*Neisseria*	*oralis oral taxon 014*	S220	Proteobacteria	Alphaproteobacteria	Caulobacterales	Caulobacteraceae	*Caulobacter*	*sp. oral taxon 002*

S13	Proteobacteria	Betaproteobacteria	Neisseriales	Neisseriaceae	*Neisseria*	*mucosa oral taxon 682*	S221	Firmicutes	Clostridia	Clostridiales	Veillonellaceae	*Veillonellaceae_[G-1]*	*sp. oral taxon 145*
S14	Bacteroidetes	Bacteroides	Bacteroidales	Porphyromonadaceae	*Porphyromonas*	*endodontalis oral taxon 273*	S222	Firmicutes	Clostridia	Clostridiales	Veillonellaceae	*Selenomonas*	*noxia oral taxon 130*
S15	Bacteroidetes	Flavobacteria	Flavobacteriales	Flavobacteriaceae	*Capnocytophaga*	*leadbetteri oral taxon 329*	S223	Firmicutes	Clostridia	Clostridiales	Peptostreptococcaceae_[XI]	*Eubacterium_[XI][G-3]*	*brachy oral taxon 557*
S16	Firmicutes	Bacilli	Lactobacillales	Streptococcaceae	*Streptococcus*	*salivarius oral taxon 755*	S224	Proteobacteria	Betaproteobacteria	Neisseriales	Neisseriaceae	*Simonsiella*	*muelleri oral taxon 683*
S17	Proteobacteria	Gammaproteobacteria	Pasteurellales	Pasteurellaceae	*Aggregatibacter*	*segnis oral taxon 762*	S225	Bacteroidetes	Bacteroides	Bacteroidales	Prevotellaceae	*Prevotella*	*scopos oral taxon 885*
S18	Firmicutes	Bacilli	Lactobacillales	Carnobacteriaceae	*Granulicatella*	*adiacens [para-adiacens] oral taxon 534*	S226	Proteobacteria	Betaproteobacteria	Burkholderiales	Comomonadaceae	*Variovorax*	*paradoxus oral taxon 717*
S19	Bacteroidetes	Bacteroides	Bacteroidales	Prevotellaceae	*Alloprevotella*	*tannerae oral taxon 466*	S227	Bacteroidetes	Bacteroides	Bacteroidales	Prevotellaceae	*Prevotella*	*oralis oral taxon 705*
S20	Actinobacteria	Actinobacteria	Actinomycetales	Micrococcaceae	*Rothia*	*aeria oral taxon 188*	S228	Firmicutes	Bacilli	Lactobacillales	Lactobacillaceae	*Lactobacillus*	*rhamnosus oral taxon 749*
S21	Bacteroidetes	Bacteroides	Bacteroidales	Prevotellaceae	*Prevotella*	*sp. oral taxon 304*	S229	Firmicutes	Clostridia	Clostridiales	Syntrophomonadaceae_[VIII]	*Syntrophomonadaceae_[VIII][G-1]*	*sp. oral taxon 435*
S22	Proteobacteria	Betaproteobacteria	Neisseriales	Neisseriaceae	*Neisseria*	*elongata oral taxon 598*	S230	Fusobacteria	Fusobacteria	Fusobacteriales	Leptotrichiaceae	*Leptotrichia*	*sp. oral taxon 219*
S23	Firmicutes	Bacilli	Lactobacillales	Streptococcaceae	*Streptococcus*	*sp. oral taxon 067*	S231	Actinobacteria	Actinobacteria	Actinomycetales	Actinomycetaceae	*Actinobaculum*	*sp. oral taxon 183*
S24	Firmicutes	Bacilli	Bacillales	Staphylococcaceae	*Gemella*	*morbillorum oral taxon 046*	S232	Bacteroidetes	Bacteroides	Bacteroidales	Prevotellaceae	*Prevotella*	*sp. oral taxon 309*
S25	Bacteroidetes	Bacteroides	Bacteroidales	Prevotellaceae	*Prevotella*	*oris oral taxon 311*	S233	Actinobacteria	Actinobacteria	Actinomycetales	Propionibacteriaceae	*Propionibacterium*	*acidifaciens oral taxon 191*

S26	Bacteroidetes	Flavobacteria	Flavobacteriales	Flavobacteriaceae	*Capnocytophaga*	*granulosa oral taxon 325*	S234	Firmicutes	Clostridia	Clostridiales	Veillonellaceae	*Megasphaera*	*sp. oral taxon 123*
S27	Bacteroidetes	Bacteroides	Bacteroidales	Prevotellaceae	*Prevotella*	*nigrescens oral taxon 693*	S235	Proteobacteria	Betaproteobacteria	Neisseriales	Neisseriaceae	*Kingella*	*sp. oral taxon 459*
S28	Bacteroidetes	Bacteroides	Bacteroidales	Prevotellaceae	*Alloprevotella*	*sp. oral taxon 914*	S236	Spirochaetes	Spirochaetes	Spirochaetales	Spirochaetaceae	*Treponema*	*pallidum oral taxon 805*
S29	Spirochaetes	Spirochaetes	Spirochaetales	Spirochaetaceae	*Treponema*	*sp. oral taxon 257*	S237	Proteobacteria	Betaproteobacteria	Neisseriales	Neisseriaceae	*Neisseria*	*lactamica oral taxon 649*
S30	Firmicutes	Bacilli	Lactobacillales	Streptococcaceae	*Streptococcus*	*sanguinis oral taxon 758*	S238	Proteobacteria	Gammaproteobacteria	Pseudomonadales	Moraxellaceae	*Moraxella*	*osloensis oral taxon 711*
S31	Actinobacteria	Actinobacteria	Actinomycetales	Actinomycetaceae	*Actinomyces*	*sp. oral taxon 180*	S239	Spirochaetes	Spirochaetes	Spirochaetales	Spirochaetaceae	*Treponema*	*sp. oral taxon 239*
S32	Proteobacteria	Betaproteobacteria	Neisseriales	Neisseriaceae	*Neisseria*	*pharyngis oral taxon 729*	S240	Firmicutes	Clostridia	Clostridiales	Peptostreptococcaceae_[XI]	*Peptostreptococcaceae_[XI][G-2]*	*sp. oral taxon 091*
S33	Spirochaetes	Spirochaetes	Spirochaetales	Spirochaetaceae	*Treponema*	*sp. oral taxon 262*	S241	Fusobacteria	Fusobacteria	Fusobacteriales	Leptotrichiaceae	*Leptotrichia*	*sp. oral taxon 218*
S34	Spirochaetes	Spirochaetes	Spirochaetales	Spirochaetaceae	*Treponema*	*sp. oral taxon 238*	S242	Bacteroidetes	Flavobacteria	Flavobacteriales	Flavobacteriaceae	*Capnocytophaga*	*sp. oral taxon 864*
S35	Bacteroidetes	Bacteroides	Bacteroidales	Prevotellaceae	*Prevotella*	*sp. oral taxon 317*	S243	Actinobacteria	Actinobacteria	Actinomycetales	Mycobacteriaceae	*Mycobacterium*	*tuberculosis oral taxon 822*
S36	Bacteroidetes	Bacteroides	Bacteroidales	Prevotellaceae	*Prevotella*	*denticola oral taxon 291*	S244	Firmicutes	Clostridia	Clostridiales	Veillonellaceae	*Dialister*	*pneumosintes oral taxon 736*
S37	Proteobacteria	Gammaproteobacteria	Pseudomonadales	Moraxellaceae	*Moraxella*	*catarrhalis oral taxon 833*	S245	Actinobacteria	Actinobacteria	Coriobacteriales	Coriobacteriaceae	*Atopobium*	*parvulum oral taxon 723*
S38	Spirochaetes	Spirochaetes	Spirochaetales	Spirochaetaceae	*Treponema*	*amylovorum oral taxon 541*	S246	Actinobacteria	Actinobacteria	Actinomycetales	Actinomycetaceae	*Actinomyces*	*massiliensis oral taxon 852*
S39	Firmicutes	Clostridia	Clostridiales	Veillonellaceae	*Selenomonas*	*sp. oral taxon 481*	S247	Fusobacteria	Fusobacteria	Fusobacteriales	Leptotrichiaceae	*Leptotrichia*	*sp. oral taxon 215*
S40	Bacteroidetes	Flavobacteria	Flavobacteriales	Flavobacteriaceae	*Capnocytophaga*	*sp. oral taxon 332*	S248	SR1	SR1_[C-1]	SR1_[O-1]	SR1_[F-1]	*SR1_[G-1]*	*sp. oral taxon 345*

S41	Fusobacteria	Fusobacteria	Fusobacteriales	Leptotrichiaceae	*Leptotrichia*	*hofstadii oral taxon 224*	S249	Spirochaetes	Spirochaetes	Spirochaetales	Spirochaetaceae	*Treponema*	*sp. oral taxon 270*
S42	Bacteroidetes	Bacteroides	Bacteroidales	Porphyromonadaceae	*Tannerella*	*sp. oral taxon 286*	S250	Proteobacteria	Betaproteobacteria	Burkholderiales	Comomonadaceae	*Delftia*	*acidovorans oral taxon 023*
S43	Bacteroidetes	Bacteroides	Bacteroidales	Prevotellaceae	*Prevotella*	*sp. oral taxon 299*	S251	Firmicutes	Erysipelotrichi	Erysipelotrichi_[O-1]	Erysipelotrichi_[F-1]	*Lactobacillus_[XVII]*	*catenaformis oral taxon 569*
S44	Proteobacteria	Betaproteobacteria	Neisseriales	Neisseriaceae	*Neisseria*	*sp. oral taxon 020*	S252	Proteobacteria	Betaproteobacteria	Burkholderiales	Comamonadaceae	*Comamonas*	*testosteroni oral taxon 858*
S45	Bacteroidetes	Bacteroides	Bacteroidales	Prevotellaceae	*Alloprevotella*	*sp. oral taxon 912*	S253	Spirochaetes	Spirochaetes	Spirochaetales	Spirochaetaceae	*Treponema*	*sp. oral taxon 255*
S46	Firmicutes	Bacilli	Lactobacillales	Streptococcaceae	*Streptococcus*	*constellatus oral taxon 576*	S254	Fusobacteria	Fusobacteria	Fusobacteriales	Leptotrichiaceae	*Leptotrichia*	*goodfellowii oral taxon 845*
S47	Synergistetes	Synergistetes_[C-1]	Synergistetes_[O-1]	Synergistetes_[F-2]	*Fretibacterium*	*sp. oral taxon 453*	S255	Proteobacteria	Alphaproteobacteria	Rhizobiales	Bradyrhizobiaceae	*Afipia*	*sp. genomospecies 8 oral taxon 636*
S48	Proteobacteria	Epsilonproteobacteria	Campylobacterales	Campylobacteraceae	*Campylobacter*	*concisus oral taxon 575*	S256	Bacteroidetes	Bacteroidetes_[C-1]	Bacteroidetes_[O-1]	Bacteroidetes_[F-1]	*Bacteroidetes_[G-5]*	*sp. oral taxon 511*
S49	Firmicutes	Clostridia	Clostridiales	Clostridiales_[F-2]	*Clostridiales_[F-2][G-1]*	*sp. oral taxon 075*	S257	TM7	TM7_[C-1]	TM7_[O-1]	TM7_[F-1]	*TM7_[G-1]*	*sp. oral taxon 346*
S50	Bacteroidetes	Bacteroides	Bacteroidales	Prevotellaceae	*Prevotella*	*sp. oral taxon 443*	S258	Firmicutes	Bacilli	Lactobacillales	Streptococcaceae	*Streptococcus*	*peroris oral taxon 728*
S51	Bacteroidetes	Bacteroides	Bacteroidales	Prevotellaceae	*Prevotella*	*sp. oral taxon 526*	S259	Proteobacteria	Betaproteobacteria	Rhodocyclales	Rhodocyclaceae	*Rhodocyclus*	*sp. oral taxon 028*
S52	Firmicutes	Clostridia	Clostridiales	Veillonellaceae	*Veillonella*	*sp. oral taxon 780*	S260	Firmicutes	Bacilli	Lactobacillales	Enterococcaceae	*Enterococcus*	*italicus oral taxon 803*
S53	Bacteroidetes	Bacteroides	Bacteroidales	Prevotellaceae	*Prevotella*	*sp. oral taxon 310*	S261	Proteobacteria	Betaproteobacteria	Neisseriales	Neisseriaceae	*Eikenella*	*corrodens oral taxon 577*
S54	Spirochaetes	Spirochaetes	Spirochaetales	Spirochaetaceae	*Treponema*	*denticola oral*	S262	Firmicutes	Bacilli	Bacillales	Bacillaceae	*Bacillus*	*anthracis oral*

						taxon 584							*taxon 824*
S55	Fusobacteria	Fusobacteria	Fusobacteriales	Fusobacteriaceae	*Fusobacterium*	*nucleatum ss vincentii oral taxon 200*	S263	Actinobacteria	Actinobacteria	Actinomycetales	Actinomycetaceae	*Actinomyces*	*sp. oral taxon 414*
S56	Actinobacteria	Actinobacteria	Actinomycetales	Actinomycetaceae	*Actinomyces*	*sp. oral taxon 177*	S264	Proteobacteria	Gammaproteobacteria	Xanthomonadales	Xanthomonadaceae	*Stenotrophomonas*	*maltophilia oral taxon 663*
S57	Firmicutes	Clostridia	Clostridiales	Peptostreptococcaceae_[XI]	*Filifactor*	*alocis oral taxon 539*	S265	Firmicutes	Clostridia	Clostridiales	Lachnospiraceae_[XIVa]	*Lachnoanaerobaculum*	*sp. oral taxon 083*
S58	Fusobacteria	Fusobacteria	Fusobacteriales	Leptotrichiaceae	*Leptotrichia*	*sp. oral taxon 462*	S266	Spirochaetes	Spirochaetes	Spirochaetales	Spirochaetaceae	*Treponema*	*sp. oral taxon 230*
S59	Proteobacteria	Epsilonproteobacteria	Campylobacterales	Campylobacteraceae	*Campylobacter*	*rectus oral taxon 748*	S267	Actinobacteria	Actinobacteria	Actinomycetales	Actinomycetaceae	*Actinomyces*	*sp. oral taxon 448*
S60	Firmicutes	Bacilli	Lactobacillales	Streptococcaceae	*Streptococcus*	*sp. oral taxon 056*	S268	Firmicutes	Clostridia	Clostridiales	Lachnospiraceae_[XIVa]	*Moryella*	*sp. oral taxon 373*
S61	Bacteroidetes	Bacteroides	Bacteroidales	Prevotellaceae	*Prevotella*	*sp. oral taxon 472*	S269	Bacteroidetes	Bacteroides	Bacteroidales	Prevotellaceae	*Prevotella*	*multisaccharivorax oral taxon 794*
S62	Fusobacteria	Fusobacteria	Fusobacteriales	Leptotrichiaceae	*Leptotrichia*	*sp. oral taxon 212*	S270	Proteobacteria	Gammaproteobacteria	Pseudomonadales	Moraxellaceae	*Acinetobacter*	*baumannii oral taxon 554*
S63	Bacteroidetes	Flavobacteria	Flavobacteriales	Flavobacteriaceae	*Capnocytophaga*	*sputigena oral taxon 775*	S271	Firmicutes	Bacilli	Lactobacillales	Streptococcaceae	*Streptococcus*	*sp. oral taxon 074*
S64	Bacteroidetes	Bacteroides	Bacteroidales	Prevotellaceae	*Prevotella*	*oulorum oral taxon 288*	S272	Spirochaetes	Spirochaetes	Spirochaetales	Spirochaetaceae	*Treponema*	*sp. oral taxon 254*
S65	Firmicutes	Clostridia	Clostridiales	Lachnospiraceae_[XIVa]	*Oribacterium*	*sp. oral taxon 108*	S273	Proteobacteria	Gammaproteobacteria	Pasteurellales	Pasteurellaceae	*Aggregatibacter*	*aphrophilus oral taxon 545*
S66	Fusobacteria	Fusobacteria	Fusobacteriales	Leptotrichiaceae	*Leptotrichia*	*sp. oral taxon 221*	S274	SR1	SR1_[C-1]	SR1_[O-1]	SR1_[F-1]	*SR1_[G-1]*	*sp. oral taxon 875*
S67	Firmicutes	Bacilli	Lactobacillales	Carnobacteriaceae	*Granulicatella*	*elegans oral taxon 596*	S275	Proteobacteria	Gammaproteobacteria	Pseudomonadales	Moraxellaceae	*Acinetobacter*	*sp. oral taxon 408*

S68	Bacteroidetes	Flavobacteria	Flavobacteriales	Flavobacteriales_[F-1]	*Flavobacteriales_[G-1]*	*sp. oral taxon 318*	S276	Bacteroidetes	Bacteroides	Bacteroidales	Porphyromonadaceae	*Porphyromonas*	*sp. oral taxon 275*
S69	Actinobacteria	Actinobacteria	Actinomycetales	Corynebacteriaceae	*Corynebacterium*	*matruchotii oral taxon 666*	S277	Bacteroidetes	Bacteroides	Bacteroidales	Prevotellaceae	*Alloprevotella*	*sp. oral taxon 308*
S70	Proteobacteria	Gammaproteobacteria	Pasteurellales	Pasteurellaceae	*Haemophilus*	*haemolyticus oral taxon 851*	S278	Spirochaetes	Spirochaetes	Spirochaetales	Spirochaetaceae	*Treponema*	*sp. oral taxon 490*
S71	Spirochaetes	Spirochaetes	Spirochaetales	Spirochaetaceae	*Treponema*	*sp. oral taxon 235*	S279	Firmicutes	Bacilli	Lactobacillales	Lactobacillaceae	*Lactobacillus*	*crispatus oral taxon 817*
S72	Firmicutes	Bacilli	Lactobacillales	Streptococcaceae	*Streptococcus*	*oligofermentans oral taxon 886*	S280	Proteobacteria	Deltaproteobacteria	Desulfobacterales	Desulfobulbaceae	*Desulfobulbus*	*sp. oral taxon 041*
S73	Actinobacteria	Actinobacteria	Coriobacteriales	Coriobacteriaceae	*Eggerthella*	*lenta oral taxon 654*	S281	Proteobacteria	Epsilonproteobacteria	Campylobacterales	Helicobacteraceae	*Helicobacter*	*pylori oral taxon 812*
S74	Bacteroidetes	Bacteroides	Bacteroidales	Prevotellaceae	*Alloprevotella*	*rava oral taxon 302*	S282	Firmicutes	Clostridia	Clostridiales	Lachnospiraceae_[XIVa]	*Oribacterium*	*sp. oral taxon 078*
S75	Firmicutes	Clostridia	Clostridiales	Veillonellaceae	*Selenomonas*	*sputigena oral taxon 151*	S283	Proteobacteria	Betaproteobacteria	Burkholderiales	Comomonadaceae	*Leptothrix*	*sp. oral taxon 025*
S76	Bacteroidetes	Bacteroides	Bacteroidales	Prevotellaceae	*Prevotella*	*maculosa oral taxon 289*	S284	Actinobacteria	Actinobacteria	Actinomycetales	Micrococcaceae	*Kocuria*	*sp. oral taxon 189*
S77	Firmicutes	Bacilli	Lactobacillales	Streptococcaceae	*Streptococcus*	*sp. oral taxon 069*	S285	Proteobacteria	Betaproteobacteria	Neisseriales	Neisseriaceae	*Kingella*	*sp. oral taxon 012*
S78	Firmicutes	Clostridia	Clostridiales	Lachnospiraceae_[XIVa]	*Catonella*	*morbi oral taxon 165*	S286	Chloroflexi	Chloroflexi_[C-1]	Chloroflexi_[O-1]	Chloroflexi_[F-1]	*Chloroflexi_[G-1]*	*sp. oral taxon 439*
S79	Proteobacteria	Gammaproteobacteria	Pasteurellales	Pasteurellaceae	*Haemophilus*	*sp. oral taxon 035*	S287	Bacteroidetes	Bacteroides	Bacteroidales	Porphyromonadaceae	*Porphyromonas*	*sp. oral taxon 285*
S80	Bacteroidetes	Bacteroides	Bacteroidales	Prevotellaceae	*Prevotella*	*pleuritidis oral taxon 303*	S288	Actinobacteria	Actinobacteria	Actinomycetales	Propionibacteriaceae	*Propionibacterium*	*acnes oral taxon 530*

S81	Fusobacteria	Fusobacteria	Fusobacteriales	Fusobacteriaceae	*Fusobacterium*	*nucleatum ss animalis oral taxon 420*	S289	Spirochaetes	Spirochaetes	Spirochaetales	Spirochaetaceae	*Treponema*	*sp. oral taxon 236*
S82	Firmicutes	Clostridia	Clostridiales	Lachnospiraceae_[XIVa]	*Lachnoanaerobaculum*	*umeaense oral taxon 107*	S290	Firmicutes	Bacilli	Lactobacillales	Streptococcaceae	*Streptococcus*	*sp. oral taxon 487*
S83	Firmicutes	Clostridia	Clostridiales	Peptostreptococcaceae_[XI]	*Peptostreptococcus*	*stomatis oral taxon 112*	S291	Actinobacteria	Actinobacteria	Coriobacteriales	Coriobacteriaceae	*Olsenella*	*sp. oral taxon 807*
S84	Bacteroidetes	Bacteroides	Bacteroidales	Prevotellaceae	*Prevotella*	*pallens oral taxon 714*	S292	Bacteroidetes	Bacteroides	Bacteroidales	Bacteroidaceae	*Bacteroides*	*zoogleoformans oral taxon 465*
S85	Bacteroidetes	Bacteroides	Bacteroidales	Prevotellaceae	*Prevotella*	*sp. oral taxon 396*	S293	Actinobacteria	Actinobacteria	Bifidobacteriales	Bifidobacteriaceae	*Scardovia*	*wiggsiae oral taxon 195*
S86	Firmicutes	Clostridia	Clostridiales	Lachnospiraceae_[XIVa]	*Moryella*	*sp. oral taxon 097*	S294	Firmicutes	Bacilli	Lactobacillales	Streptococcaceae	*Streptococcus*	*sp. oral taxon 423*
S87	Fusobacteria	Fusobacteria	Fusobacteriales	Leptotrichiaceae	*Leptotrichia*	*hongkongensis oral taxon 213*	S295	Actinobacteria	Actinobacteria	Actinomycetales	Actinomycetaceae	*Actinomyces*	*sp. oral taxon 897*
S88	Bacteroidetes	Bacteroides	Bacteroidales	Prevotellaceae	*Prevotella*	*salivae oral taxon 307*	S296	Proteobacteria	Alphaproteobacteria	Sphingomonadales	Sphingomonadaceae	*Sphingomonas*	*sp. oral taxon 006*
S89	Fusobacteria	Fusobacteria	Fusobacteriales	Leptotrichiaceae	*Sneathia*	*sanguinegens oral taxon 837*	S297	Firmicutes	Clostridia	Clostridiales	Veillonellaceae	*Selenomonas*	*sp. oral taxon 137*
S90	Bacteroidetes	Flavobacteria	Flavobacteriales	Flavobacteriaceae	*Bergeyella*	*sp. oral taxon 322*	S298	Firmicutes	Clostridia	Clostridiales	Lachnospiraceae_[XIVa]	*Oribacterium*	*sp. oral taxon 102*
S91	Actinobacteria	Actinobacteria	Actinomycetales	Corynebacteriaceae	*Corynebacterium*	*durum oral taxon 595*	S299	Firmicutes	Clostridia	Clostridiales	Lachnospiraceae_[XIVa]	*Johnsonella*	*ignava oral taxon 635*
S92	Firmicutes	Bacilli	Lactobacillales	Streptococcaceae	*Streptococcus*	*sp. oral taxon 061*	S300	Bacteroidetes	Bacteroides	Bacteroidales	Prevotellaceae	*Prevotella*	*sp. oral taxon 515*
S93	Firmicutes	Bacilli	Lactobacillales	Aerococcaceae	*Abiotrophia*	*defectiva oral taxon 389*	S301	Proteobacteria	Alphaproteobacteria	Rhodobacterales	Rhodobacteraceae	*Rhodobacter*	*capsulatus oral taxon 857*

S94	Actinobacteria	Actinobacteria	Actinomycetales	Propionibacteriaceae	*Propionibacterium*	*propionicum oral taxon 739*	S302	Proteobacteria	Betaproteobacteria	Burkholderiales	Burkholderiaceae	*Burkholderia*	*cepacia oral taxon 571*
S95	Bacteroidetes	Flavobacteria	Flavobacteriales	Flavobacteriaceae	*Capnocytophaga*	*sp. oral taxon 324*	S303	Firmicutes	Clostridia	Clostridiales	Veillonellaceae	*Mitsuokella*	*sp. oral taxon 131*
S96	Bacteroidetes	Bacteroides	Bacteroidales	Prevotellaceae	*Prevotella*	*saccharolytica oral taxon 781*	S304	Synergistetes	Synergistetes_[C-1]	Synergistetes_[O-1]	Synergistetes_[F-1]	*Pyramidobacter*	*piscolens oral taxon 357*
S97	Firmicutes	Clostridia	Clostridiales	Clostridiales_[F-2]	*Clostridiales_[F-2][G-2]*	*sp. oral taxon 085*	S305	Fusobacteria	Fusobacteria	Fusobacteriales	Leptotrichiaceae	*Leptotrichia*	*sp. oral taxon 847*
S98	Bacteroidetes	Bacteroidetes_[C-1]	Bacteroidetes_[O-1]	Bacteroidetes_[F-1]	*Bacteroidetes_[G-5]*	*sp. oral taxon 505*	S306	Tenericutes	Mollicutes	Mycoplasmatales	Mycoplasmataceae	*Mycoplasma*	*faucium oral taxon 606*
S99	Fusobacteria	Fusobacteria	Fusobacteriales	Fusobacteriaceae	*Fusobacterium*	*nucleatum ss nucleatum oral taxon 698*	S307	Actinobacteria	Actinobacteria	Actinomycetales	Actinomycetaceae	*Actinomyces*	*meyeri oral taxon 671*
S100	Bacteroidetes	Bacteroides	Bacteroidales	Bacteroidales_[F-2]	*Bacteroidales_[G-2]*	*sp. oral taxon 274*	S308	Firmicutes	Bacilli	Bacillales	Bacillaceae	*Bacillus*	*subtilis oral taxon 468*
S101	Bacteroidetes	Bacteroides	Bacteroidales	Prevotellaceae	*Prevotella*	*multiformis oral taxon 685*	S309	Proteobacteria	Betaproteobacteria	Neisseriales	Neisseriaceae	*Neisseria*	*flavescens oral taxon 610*
S102	Proteobacteria	Gammaproteobacteria	Pasteurellales	Pasteurellaceae	*Aggregatibacter*	*sp. oral taxon 898*	S310	Bacteroidetes	Flavobacteria	Flavobacteriales	Flavobacteriaceae	*Capnocytophaga*	*sp. oral taxon 902*
S103	Spirochaetes	Spirochaetes	Spirochaetales	Spirochaetaceae	*Treponema*	*sp. oral taxon 237*	S311	Proteobacteria	Betaproteobacteria	Neisseriales	Neisseriaceae	*Kingella*	*oralis oral taxon 706*
S104	Actinobacteria	Actinobacteria	Actinomycetales	Actinomycetaceae	*Actinomyces*	*graevenitzii oral taxon 866*	S312	Actinobacteria	Actinobacteria	Actinomycetales	Actinomycetaceae	*Actinomyces*	*sp. oral taxon 896*
S105	Bacteroidetes	Bacteroides	Bacteroidales	Prevotellaceae	*Prevotella*	*sp. oral taxon 293*	S313	Proteobacteria	Alphaproteobacteria	Rhizobiales	Brucellaceae	*Ochrobactrum*	*anthropi oral taxon 544*
S106	Bacteroidetes	Bacteroides	Bacteroidales	Prevotellaceae	*Prevotella*	*sp. oral taxon 313*	S314	Firmicutes	Clostridia	Clostridiales	Lachnospiraceae_[XIVa]	*Lachnoanaerobaculum*	*sp. oral taxon 089*

S107	Actinobacteria	Actinobacteria	Actinomycetales	Dietziaceae	*Dietzia*	*sp. oral taxon 368*	S315	Firmicutes	Bacilli	Lactobacillales	Lactobacillaceae	*Lactobacillus*	*salivarius oral taxon 756*
S108	Firmicutes	Clostridia	Clostridiales	Lachnospiraceae_[XIVa]	*Lachnospiraceae_[G-3]*	*sp. oral taxon 100*	S316	Firmicutes	Clostridia	Clostridiales	Peptostreptococcaceae_[XI]	*Peptostreptococcaceae_[XI][G-3]*	*sp. oral taxon 382*
S109	Firmicutes	Clostridia	Clostridiales	Veillonellaceae	*Veillonellaceae_[G-1]*	*sp. oral taxon 155*	S317	Proteobacteria	Alphaproteobacteria	Sphingomonadales	Sphingomonadaceae	*Sphingomonas*	*sp. oral taxon 007*
S110	Spirochaetes	Spirochaetes	Spirochaetales	Spirochaetaceae	*Treponema*	*sp. oral taxon 263*	S318	Proteobacteria	Alphaproteobacteria	Rhizobiales	Rhizobiaceae	*Agrobacterium*	*tumefaciens oral taxon 485*
S111	Bacteroidetes	Flavobacteria	Flavobacteriales	Flavobacteriaceae	*Capnocytophaga*	*sp. oral taxon 326*	S319	Firmicutes	Bacilli	Bacillales	Staphylococcaceae	*Staphylococcus*	*caprae oral taxon 567*
S112	Bacteroidetes	Bacteroides	Bacteroidales	Porphyromonadaceae	*Tannerella*	*forsythia oral taxon 613*	S320	Firmicutes	Clostridia	Clostridiales	Veillonellaceae	*Veillonella*	*rogosae oral taxon 158*
S113	Firmicutes	Clostridia	Clostridiales	Peptostreptococcaceae_[XI]	*Eubacterium_[XI][G-5]*	*saphenum oral taxon 759*	S321	Spirochaetes	Spirochaetes	Spirochaetales	Spirochaetaceae	*Treponema*	*parvum oral taxon 724*
S114	Bacteroidetes	Bacteroides	Bacteroidales	Prevotellaceae	*Prevotella*	*dentalis oral taxon 583*	S322	Bacteroidetes	Flavobacteria	Flavobacteriales	Flavobacteriaceae	*Capnocytophaga*	*sp. oral taxon 336*
S115	Proteobacteria	Epsilonproteobacteria	Campylobacterales	Campylobacteraceae	*Campylobacter*	*sp. oral taxon 044*	S323	Actinobacteria	Actinobacteria	Actinomycetales	Corynebacteriaceae	*Corynebacterium*	*diphtheriae oral taxon 591*
S116	Bacteroidetes	Bacteroides	Bacteroidales	Prevotellaceae	*Prevotella*	*veroralis oral taxon 572*	S324	Fusobacteria	Fusobacteria	Fusobacteriales	Leptotrichiaceae	*Leptotrichia*	*shahii oral taxon 214*
S117	Spirochaetes	Spirochaetes	Spirochaetales	Spirochaetaceae	*Treponema*	*sp. oral taxon 234*	S325	Actinobacteria	Actinobacteria	Actinomycetales	Propionibacteriaceae	*Propionibacterium*	*sp. oral taxon 915*
S118	Bacteroidetes	Bacteroides	Bacteroidales	Prevotellaceae	*Prevotella*	*micans oral taxon 378*	S326	TM7	TM7_[C-1]	TM7_[O-1]	TM7_[F-1]	*TM7_[G-1]*	*sp. oral taxon 352*
S119	Bacteroidetes	Flavobacteria	Flavobacteriales	Flavobacteriaceae	*Bergeyella*	*sp. oral taxon 900*	S327	Proteobacteria	Betaproteobacteria	Burkholderiales	Ralstoniaceae	*Ralstonia*	*pickettii oral taxon 854*

S120	Firmicutes	Clostridia	Clostridiales	Clostridiales_[F-1]	*Clostridiales_[F-1][G-1]*	*sp. oral taxon 093*	S328	Firmicutes	Bacilli	Lactobacillales	Streptococcaceae	*Lactococcus*	*lactis oral taxon 804*
S121	Firmicutes	Clostridia	Clostridiales	Lachnospiraceae_[XIVa]	*Shuttleworthia*	*satelles oral taxon 095*	S329	Synergistetes	Synergistetes_[C-1]	Synergistetes_[O-1]	Synergistetes_[F-1]	*Jonquetella*	*anthropi oral taxon 777*
S122	Fusobacteria	Fusobacteria	Fusobacteriales	Leptotrichiaceae	*Leptotrichiaceae_[G-1]*	*sp. oral taxon 210*	S330	Firmicutes	Clostridia	Clostridiales	Lachnospiraceae_[XIVa]	*Lachnoanaerobaculum*	*sp. oral taxon 496*
S123	Synergistetes	Synergistetes_[C-1]	Synergistetes_[O-1]	Synergistetes_[F-2]	*Fretibacterium*	*fastidiosum oral taxon 363*	S331	Proteobacteria	Gammaproteobacteria	Pseudomonadales	Pseudomonadaceae	*Pseudomonas*	*pseudoalcaligenes oral taxon 740*
S124	Bacteroidetes	Bacteroidetes_[C-1]	Bacteroidetes_[O-1]	Bacteroidetes_[F-1]	*Bacteroidetes_[G-3]*	*sp. oral taxon 503*	S332	Actinobacteria	Actinobacteria	Actinomycetales	Actinomycetaceae	*Actinomyces*	*sp. oral taxon 172*
S125	Bacteroidetes	Bacteroides	Bacteroidales	Prevotellaceae	*Prevotella*	*shahii oral taxon 795*	S333	Fusobacteria	Fusobacteria	Fusobacteriales	Fusobacteriaceae	*Fusobacterium*	*sp. oral taxon 205*
S126	Bacteroidetes	Bacteroides	Bacteroidales	Prevotellaceae	*Prevotella*	*histicola oral taxon 298*	S334	Firmicutes	Clostridia	Clostridiales	Veillonellaceae	*Selenomonas*	*dianae oral taxon 139*
S127	Bacteroidetes	Flavobacteria	Flavobacteriales	Flavobacteriaceae	*Capnocytophaga*	*sp. oral taxon 901*	S335	Proteobacteria	Alphaproteobacteria	Rhizobiales	Rhizobiaceae	*Rhizobium*	*loti oral taxon 659*
S128	Proteobacteria	Gammaproteobacteria	Pasteurellales	Pasteurellaceae	*Haemophilus*	*parainfluenzae oral taxon 718*	S336	Actinobacteria	Actinobacteria	Actinomycetales	Actinomycetaceae	*Actinomyces*	*georgiae oral taxon 617*
S129	Fusobacteria	Fusobacteria	Fusobacteriales	Fusobacteriaceae	*Fusobacterium*	*sp. oral taxon 203*	S337	Firmicutes	Bacilli	Lactobacillales	Streptococcaceae	*Streptococcus*	*sp. oral taxon 055*
S130	Proteobacteria	Gammaproteobacteria	Pasteurellales	Pasteurellaceae	*Aggregatibacter*	*sp. oral taxon 458*	S338	Proteobacteria	Gammaproteobacteria	Pasteurellales	Pasteurellaceae	*Aggregatibacter*	*paraphrophilus oral taxon 720*
S131	Bacteroidetes	Bacteroides	Bacteroidales	Prevotellaceae	*Prevotella*	*buccae oral taxon 560*	S339	Firmicutes	Clostridia	Clostridiales	Lachnospiraceae_[XIVa]	*Lachnospiraceae_[G-5]*	*sp. oral taxon 455*
S132	Bacteroidetes	Bacteroides	Bacteroidales	Bacteroidaceae	*Bacteroidaceae_[G-1]*	*sp. oral taxon 272*	S340	Actinobacteria	Actinobacteria	Bifidobacteriales	Bifidobacteriaceae	*Scardovia*	*inopinata oral taxon 642*
S133	Spirochaetes	Spirochaetes	Spirochaetales	Spirochaetaceae	*Treponema*	*socranskii oral*	S341	Actinobacteria	Actinobacteria	Actinomycetales	Actinomycetaceae	*Actinomyces*	*timonensis oral*

						taxon 769							*taxon 179*
S134	Spirochaetes	Spirochaetes	Spirochaetales	Spirochaetaceae	*Treponema*	*sp. oral taxon 258*	S342	Actinobacteria	Actinobacteria	Actinomycetales	Actinomycetaceae	*Actinomyces*	*oricola oral taxon 708*
S135	Proteobacteria	Betaproteobacteria	Burkholderiales	Comamonadaceae	*Ottowia*	*sp. oral taxon 894*	S343	Proteobacteria	Gammaproteobacteria	Pseudomonadales	Pseudomonadaceae	*Pseudomonas*	*stutzeri oral taxon 477*
S136	Bacteroidetes	Bacteroides	Bacteroidales	Prevotellaceae	*Prevotella*	*baroniae oral taxon 553*	S344	Spirochaetes	Spirochaetes	Spirochaetales	Spirochaetaceae	*Treponema*	*sp. oral taxon 227*
S137	Firmicutes	Clostridia	Clostridiales	Peptostreptococcaceae_[XIII]	*Parvimonas*	*micra oral taxon 111*	S345	Actinobacteria	Actinobacteria	Actinomycetales	Actinomycetaceae	*Actinomyces*	*radicidentis oral taxon 746*
S138	Bacteroidetes	Bacteroides	Bacteroidales	Bacteroidaceae	*Bacteroides*	*heparinolyticus oral taxon 630*	S346	Firmicutes	Bacilli	Lactobacillales	Streptococcaceae	*Streptococcus*	*mutans oral taxon 686*
S139	Bacteroidetes	Flavobacteria	Flavobacteriales	Flavobacteriaceae	*Bergeyella*	*sp. oral taxon 907*	S347	Bacteroidetes	Flavobacteria	Flavobacteriales	Flavobacteriales_[F-2]	*Flavobacteriales_[G-2]*	*sp. oral taxon 320*
S140	Spirochaetes	Spirochaetes	Spirochaetales	Spirochaetaceae	*Treponema*	*sp. oral taxon 256*	S348	Proteobacteria	Gammaproteobacteria	Pasteurellales	Pasteurellaceae	*Haemophilus*	*influenzae oral taxon 641*
S141	Bacteroidetes	Bacteroidetes_[C-2]	Bacteroidetes_[O-2]	Bacteroidetes_[F-2]	*Bacteroidetes_[G-6]*	*sp. oral taxon 516*	S349	Proteobacteria	Gammaproteobacteria	Pseudomonadales	Pseudomonadaceae	*Pseudomonas*	*fluorescens oral taxon 612*
S142	Firmicutes	Clostridia	Clostridiales	Peptostreptococcaceae_[XI]	*Eubacterium_[XI][G-6]*	*nodatum oral taxon 694*	S350	Fusobacteria	Fusobacteria	Fusobacteriales	Leptotrichiaceae	*Leptotrichia*	*sp. oral taxon 909*
S143	Spirochaetes	Spirochaetes	Spirochaetales	Spirochaetaceae	*Treponema*	*vincentii oral taxon 029*	S351	Proteobacteria	Deltaproteobacteria	Desulfovibrionales	Desulfovibrionaceae	*Desulfovibrio*	*sp. oral taxon 040*
S144	Bacteroidetes	Bacteroidetes_[C-1]	Bacteroidetes_[O-1]	Bacteroidetes_[F-1]	*Bacteroidetes_[G-3]*	*sp. oral taxon 365*	S352	TM7	TM7_[C-1]	TM7_[O-1]	TM7_[F-1]	*TM7_[G-4]*	*sp. oral taxon 355*
S145	GN02	GN02_[C-1]	GN02_[O-1]	GN02_[F-1]	*GN02_[G-1]*	*sp. oral taxon 872*	S353	Bacteroidetes	Bacteroidetes_[C-1]	Bacteroidetes_[O-1]	Bacteroidetes_[F-1]	*Bacteroidetes_[G-5]*	*sp. oral taxon 507*
S146	Firmicutes	Clostridia	Clostridiales	Veillonellaceae	*Dialister*	*invisus oral taxon*	S354	Bacteroidetes	Bacteroidetes_[C-	Bacteroidetes_[O-	Bacteroidetes_[F-	*Bacteroidetes_[G-*	*sp. oral taxon 509*

						118			1]	1]	1]	*4]*	
S147	Proteobacteria	Gammaproteobacteria	Pasteurellales	Pasteurellaceae	*Haemophilus*	*sp. oral taxon 036*	S355	Proteobacteria	Betaproteobacteria	Neisseriales	Neisseriaceae	*Neisseria*	*meningitidis oral taxon 669*
S148	Firmicutes	Clostridia	Clostridiales	Peptostreptococcaceae_[XI]	*Mogibacterium*	*timidum oral taxon 042*	S356	Firmicutes	Clostridia	Clostridiales	Veillonellaceae	*Selenomonas*	*flueggei oral taxon 125*
S149	Firmicutes	Bacilli	Lactobacillales	Lactobacillaceae	*Lactobacillus*	*vaginalis oral taxon 051*	S357	Proteobacteria	Alphaproteobacteria	Rhizobiales	Bradyrhizobiaceae	*Bradyrhizobium*	*elkanii oral taxon 597*
S150	Fusobacteria	Fusobacteria	Fusobacteriales	Leptotrichiaceae	*Leptotrichia*	*sp. oral taxon 498*	S358	Bacteroidetes	Flavobacteria	Flavobacteriales	Flavobacteriaceae	*Capnocytophaga*	*ochracea oral taxon 700*
S151	Bacteroidetes	Flavobacteria	Flavobacteriales	Flavobacteriaceae	*Capnocytophaga*	*sp. oral taxon 878*	S359	Proteobacteria	Gammaproteobacteria	Pseudomonadales	Pseudomonadaceae	*Pseudomonas*	*aeruginosa oral taxon 536*
S152	Firmicutes	Clostridia	Clostridiales	Lachnospiraceae_[XIVa]	*Catonella*	*sp. oral taxon 451*	S360	Firmicutes	Clostridia	Clostridiales	Eubacteriaceae_[XV]	*Pseudoramibacter*	*alactolyticus oral taxon 538*
S153	Firmicutes	Clostridia	Clostridiales	Peptostreptococcaceae_[XI]	*Eubacterium_[XI][G-1]*	*sulci oral taxon 467*	S361	Firmicutes	Clostridia	Clostridiales	Peptostreptococcaceae_[XIII]	*Parvimonas*	*sp oral taxon 110*
S154	Proteobacteria	Gammaproteobacteria	Cardiobacteriales	Cardiobacteriaceae	*Cardiobacterium*	*hominis oral taxon 633*	S362	Firmicutes	Clostridia	Clostridiales	Peptostreptococcaceae_[XIII]	*Peptostreptococcaceae_[XIII][G-1]*	*sp. oral taxon 113*
S155	Firmicutes	Clostridia	Clostridiales	Lachnospiraceae_[XIVa]	*Lachnospiraceae_[G-8]*	*sp. oral taxon 500*	S363	Firmicutes	Clostridia	Clostridiales	Veillonellaceae	*Mitsuokella*	*multacida oral taxon 684*
S156	Spirochaetes	Spirochaetes	Spirochaetales	Spirochaetaceae	*Treponema*	*sp. oral taxon 231*	S364	Actinobacteria	Actinobacteria	Coriobacteriales	Coriobacteriaceae	*Atopobium*	*sp oral taxon 810*
S157	TM7	TM7_[C-1]	TM7_[O-1]	TM7_[F-1]	*TM7_[G-2]*	*sp. oral taxon 350*	S365	Actinobacteria	Actinobacteria	Actinomycetales	Actinomycetaceae	*Mobiluncus*	*mulieris oral taxon 830*
S158	Fusobacteria	Fusobacteria	Fusobacteriales	Leptotrichiaceae	*Leptotrichia*	*buccalis oral taxon 563*	S366	Firmicutes	Bacilli	Lactobacillales	Lactobacillaceae	*Lactobacillus*	*plantarum oral taxon 861*
S159	Bacteroidetes	Bacteroidetes_[C-1]	Bacteroidetes_[O-1]	Bacteroidetes_[F-1]	*Bacteroidetes_[G-3]*	*sp. oral taxon 280*	S367	Actinobacteria	Actinobacteria	Coriobacteriales	Coriobacteriaceae	*Atopobium*	*sp. oral taxon 416*

S160	Actinobacteria	Actinobacteria	Actinomycetales	Actinomycetaceae	*Actinomyces*	*sp. oral taxon 848*	S368	Firmicutes	Clostridia	Clostridiales	Lachnospiraceae_[XIVa]	*Eubacterium_[XIVa][G-1]*	*saburreum oral taxon 494*
S161	Bacteroidetes	Flavobacteria	Flavobacteriales	Flavobacteriaceae	*Capnocytophaga*	*gingivalis oral taxon 337*	S369	Firmicutes	Clostridia	Clostridiales	Veillonellaceae	*Selenomonas*	*sp. oral taxon 388*
S162	Fusobacteria	Fusobacteria	Fusobacteriales	Leptotrichiaceae	*Leptotrichia*	*sp. oral taxon 392*	S370	Bacteroidetes	Flavobacteria	Flavobacteriales	Flavobacteriaceae	*Bergeyella*	*sp. oral taxon 319*
S163	Proteobacteria	Deltaproteobacteria	Desulfovibrionales	Desulfovibrionaceae	*Desulfovibrio*	*fairfieldensis oral taxon 605*	S371	GN02	GN02_[C-1]	GN02_[O-1]	GN02_[F-1]	*GN02_[G-1]*	*sp. oral taxon 871*
S164	Firmicutes	Clostridia	Clostridiales	Peptococcaceae	*Peptococcus*	*sp. oral taxon 167*	S372	Proteobacteria	Betaproteobacteria	Neisseriales	Neisseriaceae	*Eikenella*	*sp. oral taxon 011*
S165	Bacteroidetes	Flavobacteria	Flavobacteriales	Flavobacteriaceae	*Capnocytophaga*	*sp. oral taxon 903*	S373	Bacteroidetes	Flavobacteria	Flavobacteriales	Flavobacteriaceae	*Capnocytophaga*	*haemolytica oral taxon 627*
S166	Bacteroidetes	Bacteroides	Bacteroidales	Prevotellaceae	*Prevotella*	*sp. oral taxon 475*	S374	Actinobacteria	Actinobacteria	Coriobacteriales	Coriobacteriaceae	*Cryptobacterium*	*curtum oral taxon 579*
S167	Bacteroidetes	Bacteroides	Bacteroidales	Prevotellaceae	*Prevotella*	*fusca oral taxon 782*	S375	Proteobacteria	Betaproteobacteria	Burkholderiales	Ralstoniaceae	*Ralstonia*	*sp. oral taxon 027*
S168	Firmicutes	Clostridia	Clostridiales	Veillonellaceae	*Megasphaera*	*micronuciformis oral taxon 122*	S376	Firmicutes	Bacilli	Lactobacillales	Lactobacillaceae	*Lactobacillus*	*fermentum oral taxon 608*
S169	Firmicutes	Clostridia	Clostridiales	Veillonellaceae	*Veillonella*	*dispar oral taxon 160*	S377	Actinobacteria	Actinobacteria	Coriobacteriales	Coriobacteriaceae	*Slackia*	*exigua oral taxon 602*
S170	Spirochaetes	Spirochaetes	Spirochaetales	Spirochaetaceae	*Treponema*	*lecithinolyticum oral taxon 653*	S378	Proteobacteria	Betaproteobacteria	Burkholderiales	Burkholderiaceae	*Ralstonia*	*sp. oral taxon 406*
S171	Bacteroidetes	Flavobacteria	Flavobacteriales	Flavobacteriaceae	*Capnocytophaga*	*sp. oral taxon 412*	S379	Actinobacteria	Actinobacteria	Coriobacteriales	Coriobacteriaceae	*Olsenella*	*uli oral taxon 038*
S172	Spirochaetes	Spirochaetes	Spirochaetales	Spirochaetaceae	*Treponema*	*maltophilum oral taxon 664*	S380	Spirochaetes	Spirochaetes	Spirochaetales	Spirochaetaceae	*Treponema*	*sp. oral taxon 264*
S173	Bacteroidetes	Bacteroides	Bacteroidales	Prevotellaceae	*Prevotella*	*enoeca oral taxon 600*	S381	Proteobacteria	Alphaproteobacteria	Rhizobiales	Bradyrhizobiaceae	*Afipia*	*broomeae oral taxon 559*

S174	Bacteroidetes	Bacteroides	Bacteroidales	Prevotellaceae	*Prevotella*	*bivia oral taxon 556*	S382	Firmicutes	Bacilli	Lactobacillales	Streptococcaceae	*Streptococcus*	*sobrinus oral taxon 768*
S175	Bacteroidetes	Bacteroides	Bacteroidales	Prevotellaceae	*Prevotella*	*sp. oral taxon 315*	S383	Tenericutes	Mollicutes	Mollicutes_[O-1]	Mollicutes_[F-1]	*Mollicutes_[G-1]*	*sp. oral taxon 504*
S176	Proteobacteria	Deltaproteobacteria	Desulfovibrionales	Desulfomicrobiaceae	*Desulfomicrobium*	*orale oral taxon 703*	S384	Proteobacteria	Gammaproteobacteria	Pseudomonadales	Pseudomonadaceae	*Pseudomonas*	*sp. oral taxon 032*
S177	Fusobacteria	Fusobacteria	Fusobacteriales	Leptotrichiaceae	*Leptotrichia*	*sp. oral taxon 879*	S385	Proteobacteria	Gammaproteobacteria	Enterobacteriales	Enterobacteriaceae	*Escherichia*	*coli oral taxon 574*
S178	Firmicutes	Clostridia	Clostridiales	Veillonellaceae	*Veillonellaceae_[G-1]*	*sp. oral taxon 129*	S386	Proteobacteria	Alphaproteobacteria	Sphingomonadales	Sphingomonadaceae	*Erythromicrobium*	*ramosum oral taxon 747*
S179	Spirochaetes	Spirochaetes	Spirochaetales	Spirochaetaceae	*Treponema*	*sp. oral taxon 249*	S387	Firmicutes	Bacilli	Lactobacillales	Enterococcaceae	*Enterococcus*	*durans oral taxon 880*
S180	Bacteroidetes	Bacteroidetes_[C-1]	Bacteroidetes_[O-1]	Bacteroidetes_[F-1]	*Bacteroidetes_[G-3]*	*sp. oral taxon 436*	S388	Actinobacteria	Actinobacteria	Coriobacteriales	Coriobacteriaceae	*Olsenella*	*profusa oral taxon 806*
S181	Firmicutes	Clostridia	Clostridiales	Lachnospiraceae_[XIVa]	*Lachnospiraceae_[G-2]*	*sp. oral taxon 088*	S389	Bacteroidetes	Bacteroides	Bacteroidales	Porphyromonadaceae	*Tannerella*	*sp. oral taxon 916*
S182	Firmicutes	Bacilli	Lactobacillales	Streptococcaceae	*Streptococcus*	*downei oral taxon 594*	S390	Proteobacteria	Betaproteobacteria	Burkholderiales	Alcaligenaceae	*Achromobacter*	*xylosoxidans oral taxon 343*
S183	Actinobacteria	Actinobacteria	Coriobacteriales	Coriobacteriaceae	*Atopobium*	*rimae oral taxon 750*	S391	Actinobacteria	Actinobacteria	Actinomycetales	Corynebacteriaceae	*Corynebacterium*	*mucifaciens oral taxon 835*
S184	Bacteroidetes	Bacteroides	Bacteroidales	Prevotellaceae	*Prevotella*	*marshii oral taxon 665*	S392	TM7	TM7_[C-1]	TM7_[O-1]	TM7_[F-2]	*TM7_[G-5]*	*sp. oral taxon 356*
S185	Proteobacteria	Betaproteobacteria	Neisseriales	Neisseriaceae	*Neisseria*	*sicca oral taxon 764*	S393	Firmicutes	Bacilli	Lactobacillales	Lactobacillaceae	*Lactobacillus*	*paracasei oral taxon 716*
S186	Firmicutes	Clostridia	Clostridiales	Lachnospiraceae_[XIVa]	*Lachnoanaerobaculum*	*orale oral taxon 082*	S394	Firmicutes	Bacilli	Lactobacillales	Lactobacillaceae	*Lactobacillus*	*gasseri oral taxon 615*
S187	Spirochaetes	Spirochaetes	Spirochaetales	Spirochaetaceae	*Treponema*	*sp. oral taxon 250*	S395	Firmicutes	Clostridia	Clostridiales	Peptostreptococca	*Finegoldia*	*magna oral taxon*

											ceae_[XIII]		*662*
S188	Firmicutes	Clostridia	Clostridiales	Lachnospiraceae_[XIVa]	*Johnsonella*	*sp. oral taxon 166*	S396	Fusobacteria	Fusobacteria	Fusobacteriales	Fusobacteriaceae	*Fusobacterium*	*gonidiaformans oral taxon 860*
S189	Firmicutes	Clostridia	Clostridiales	Peptostreptococcaceae_[XI]	*Peptostreptococcaceae_[XI][G-4]*	*sp. oral taxon 369*	S397	TM7	TM7_[C-1]	TM7_[O-1]	TM7_[F-1]	*TM7_[G-1]*	*sp. oral taxon 349*
S190	Bacteroidetes	Bacteroides	Bacteroidales	Prevotellaceae	*Alloprevotella*	*sp. oral taxon 474*	S398	Spirochaetes	Spirochaetes	Spirochaetales	Spirochaetaceae	*Treponema*	*sp. oral taxon 518*
S191	Firmicutes	Clostridia	Clostridiales	Veillonellaceae	*Selenomonas*	*sp. oral taxon 478*	S399	Proteobacteria	Alphaproteobacteria	Sphingomonadales	Sphingomonadaceae	*Sphingomonas*	*sp. oral taxon 004*
S192	Spirochaetes	Spirochaetes	Spirochaetales	Spirochaetaceae	*Treponema*	*sp. oral taxon 517*	S400	Firmicutes	Bacilli	Lactobacillales	Lactobacillaceae	*Lactobacillus*	*panis oral taxon 882*
S193	Spirochaetes	Spirochaetes	Spirochaetales	Spirochaetaceae	*Treponema*	*sp. oral taxon 265*	S401	Proteobacteria	Gammaproteobacteria	Pasteurellales	Pasteurellaceae	*Aggregatibacter*	*actinomycetemcomitans oral taxon 531*
S194	Bacteroidetes	Flavobacteria	Flavobacteriales	Flavobacteriaceae	*Capnocytophaga*	*sp. oral taxon 863*	S402	Proteobacteria	Gammaproteobacteria	Enterobacteriales	Enterobacteriaceae	*Klebsiella*	*pneumoniae oral taxon 731*
S195	Proteobacteria	Epsilonproteobacteria	Campylobacterales	Campylobacteraceae	*Campylobacter*	*gracilis oral taxon 623*	S403	Firmicutes	Clostridia	Clostridiales	Peptostreptococcaceae_[XI]	*Eubacterium_[XI][G-1]*	*infirmum oral taxon 105*
S196	Bacteroidetes	Bacteroides	Bacteroidales	Prevotellaceae	*Prevotella*	*sp. oral taxon 301*	S404	Bacteroidetes	Bacteroidetes_[C-1]	Bacteroidetes_[O-1]	Bacteroidetes_[F-1]	*Bacteroidetes_[G-3]*	*sp. oral taxon 899*
S197	Spirochaetes	Spirochaetes	Spirochaetales	Spirochaetaceae	*Treponema*	*sp. oral taxon 246*	S405	Bacteroidetes	Flavobacteria	Flavobacteriales	Flavobacteriales_[F-1]	*Flavobacteriales_[G-1]*	*sp. oral taxon 321*
S198	Firmicutes	Clostridia	Clostridiales	Peptostreptococcaceae_[XI]	*Peptostreptococcaceae_[XI][G-5]*	*sp. oral taxon 493*	S406	Actinobacteria	Actinobacteria	Actinomycetales	Actinomycetaceae	*Arcanobacterium*	*haemolyticum oral taxon 811*
S199	Proteobacteria	Betaproteobacteria	Neisseriales	Neisseriaceae	*Neisseria*	*sp. oral taxon 016*	S407	Spirochaetes	Spirochaetes	Spirochaetales	Spirochaetaceae	*Treponema*	*pectinovorum oral taxon 725*
S200	Spirochaetes	Spirochaetes	Spirochaetales	Spirochaetaceae	*Treponema*	*sp. oral taxon 226*	S408	Actinobacteria	Actinobacteria	Bifidobacteriales	Bifidobacteriacea	*Bifidobacterium*	*dentium oral*

											e		*taxon 588*
S201	Actinobacteria	Actinobacteria	Actinomycetales	Actinomycetaceae	*Actinomyces*	*israelii oral taxon 645*	S409	Actinobacteria	Actinobacteria	Actinomycetales	Microbacteriaceae	*Microbacterium*	*sp. oral taxon 186*
S202	Firmicutes	Clostridia	Clostridiales	Peptostreptococcaceae_[XIII]	*Peptostreptococcaceae_[XIII][G-2]*	*sp. oral taxon 790*	S410	Proteobacteria	Gammaproteobacteria	Enterobacteriales	Enterobacteriaceae	*Enterobacter*	*sakazakii oral taxon 753*
S203	Fusobacteria	Fusobacteria	Fusobacteriales	Fusobacteriaceae	*Fusobacterium*	*sp. oral taxon 370*	S411	Bacteroidetes	Flavobacteria	Flavobacteriales	Flavobacteriaceae	*Capnocytophaga*	*sp. oral taxon 338*
S204	Actinobacteria	Actinobacteria	Actinomycetales	Actinomycetaceae	*Actinomyces*	*dentalis oral taxon 888*	S412	Firmicutes	Bacilli	Lactobacillales	Enterococcaceae	*Enterococcus*	*saccharolyticus oral taxon 802*
S205	Firmicutes	Bacilli	Bacillales	Bacillaceae	*Lysinibacillus*	*fusiformis oral taxon 614*	S413	Proteobacteria	Gammaproteobacteria	Enterobacteriales	Enterobacteriaceae	*Yersinia*	*pestis oral taxon 827*
S206	Firmicutes	Clostridia	Clostridiales	Peptostreptococcaceae_[XI]	*Peptostreptococcaceae_[XI][G-1]*	*sp. oral taxon 383*	S414	Firmicutes	Bacilli	Lactobacillales	Streptococcaceae	*Streptococcus*	*agalactiae oral taxon 537*
S207	Firmicutes	Clostridia	Clostridiales	Veillonellaceae	*Mitsuokella*	*sp. oral taxon 521*	S415	Proteobacteria	Alphaproteobacteria	Rhizobiales	Bartonellaceae	*Bartonella*	*sp. oral taxon 001*
S208	Proteobacteria	Alphaproteobacteria	Caulobacterales	Caulobacteraceae	*Brevundimonas*	*diminuta oral taxon 590*							

Reference

1. **Zhou JY, Jiang N, Wang SG, Hu XP, Jiao KL, He XY, Li ZQ, Wang JZ.** 2016. Exploration of Human Salivary Microbiomes-Insights into the Novel Characteristics of Microbial Community Structure in Caries and Caries-Free Subjects. Plos One **11**.
2. **Holgerson PL, Ouml;hman C, Ronnlund A, Johansson I.** 2015. Maturation of Oral Microbiota in Children with or without Dental Caries. Plos One **10**.
3. **Chen L, Qin B, Du M, Zhong H, Xu Q, Li Y, Zhang P, Fan M.** 2015. Extensive description and comparison of human supra-gingival microbiome in root caries and health. PLoS One **10:**e0117064.
4. **Xu H, Hao WJ, Zhou Q, Wang WH, Xia ZK, Liu C, Chen XC, Qin M, Chen F.** 2014. Plaque Bacterial Microbiome Diversity in Children Younger than 30 Months with or without Caries Prior to Eruption of Second Primary Molars. Plos One **9**.
5. **Jiang W, Ling ZX, Lin XL, Chen YD, Zhang J, Yu JJ, Xiang C, Chen H.** 2014. Pyrosequencing Analysis of Oral Microbiota Shifting in Various Caries States in Childhood. Microbial Ecology **67:**962-969.
6. **Belstrom D, Fiehn NE, Nielsen CH, Holmstrup P, Kirkby N, Klepac-Ceraj V, Paster BJ, Twetman S.** 2014. Altered Bacterial Profiles in Saliva from Adults with Caries Lesions: A Case-Cohort Study. Caries Research **48:**368-375.
7. **Tao Y, Zhou Y, Ouyang Y, Lin H.** 2013. Dynamics of oral microbial community profiling during severe early childhood caries development monitored by PCR-DGGE. Arch Oral Biol **58:**1129-1138.
8. **Luo AH, Yang DQ, Xin BC, Paster BJ, Qin J.** 2012. Microbial profiles in saliva from children with and without caries in mixed dentition. Oral Diseases **18:**595-601.
9. **Yang F, Zeng XW, Ning K, Liu KL, Lo CC, Wang W, Chen J, Wang DM, Huang RR, Chang XZ, Chain PS, Xie G, Ling JQ, JianXu.** 2012. Saliva microbiomes distinguish caries-active from healthy human populations. Isme Journal **6:**1-10.
10. **Tanner ACR, Mathney JMJ, Kent RL, Chalmers NI, Hughes CV, Loo CY, Pradhan N, Kanasi E, Hwang J, Dahlan MA, Papadopolou E, Dewhirst FE.** 2011. Cultivable Anaerobic Microbiota of Severe Early Childhood Caries. Journal of Clinical Microbiology **49:**1464-1474.
11. **Gross EL, Leys EJ, Gasparovich SR, Firestone ND, Schwartzbaum JA, Janies DA, Asnani K, Griffen AL.** 2010. Bacterial 16S sequence analysis of severe caries in young permanent teeth. J Clin Microbiol **48:**4121-4128.
12. **Kanasi E, Dewhirst FE, Chalmers NI, Kent R, Moore A, Hughes CV, Pradhan N, Loo CY, Tanner ACR.** 2010. Clonal Analysis of the Microbiota of Severe Early Childhood Caries. Caries Research **44:**485-497.
13. **Ling ZX, Kong JM, Jia P, Wei CC, Wang YZ, Pan ZW, Huang WJ, Li LJ, Chen H, Xiang C.** 2010. Analysis of Oral Microbiota in Children with Dental Caries by PCR-DGGE and Barcoded Pyrosequencing. Microbial Ecology **60:**677-690.
14. **Li Y, Ge Y, Saxena D, Caufield PW.** 2007. Genetic profiling of the oral microbiota associated with severe early-childhood caries. J Clin Microbiol **45:**81-87.

15. **Li Y, Ku CYS, Xu J, Saxena D, Caufield PW.** 2005. Survey of oral microbial diversity using PCR-based denaturing gradient gel electrophoresis. Journal of Dental Research **84:**559-564.
